# A synthesis of economic data from randomized trials of recipient-focused interventions to increase vaccine uptake

**DOI:** 10.1016/j.vaccine.2025.127906

**Published:** 2025-11-03

**Authors:** Yuri Hamashima, Katie Breheny, Sarah R. Davies, Sarah Dawson, Zak A. Thornton, Elisabeth Aiton, Deborah M. Caldwell, Hannah Christensen, Julian P.T. Higgins, Julie Yates, Louise Letley, Clare E. French

**Affiliations:** a NIHR Applied Research Collaboration West (ARC West) at University Hospitals Bristol and Weston NHS Foundation Trust, Bristol, United Kingdom; b Population Health Sciences, Bristol Medical School, University of Bristol, Bristol, United Kingdom; c NIHR Health Protection Research Unit in Evaluation and Behavioural Science, University of Bristol, Bristol, United Kingdom; d MRC Integrative Epidemiology Unit, University of Bristol, Bristol, United Kingdom; e UK Health Security Agency, United Kingdom

**Keywords:** Vaccine uptake, public health, economics of vaccination, systematic review

## Abstract

**Introduction:**

Achieving high vaccine uptake is key to maximizing the effectiveness of immunization programmes. Decisions around which interventions to implement to increase vaccine uptake need to be informed by both intervention effectiveness and the economic data. We aimed to collate cost analyses and economic evaluations of recipient-focused interventions to increase vaccine uptake for high and upper-middle income countries.

**Methods:**

We conducted a review of the economic evidence, alongside a systematic review of randomized controlled trials (RCTs) on the effectiveness of interventions to increase vaccine uptake. Eligible studies were those reporting economic data (including economic evaluations or cost analyses) pertaining to RCTs included in the systematic review. These studies were identified in two ways: firstly, through the comprehensive systematic review searches (up to April 2024), and secondly, through a targeted search for economic information conducted on MEDLINE to March 2024 and the archived NHS Economic Evaluation Database (EED). The quality of economic evaluations was assessed using the Drummond checklist. Data were narratively synthesized.

**Results:**

We screened 2803 reports in total, of which 37 studies met our eligibility criteria. Sixteen conducted full economic evaluations and ten conducted cost analyses, while the remaining ten reported the value of financial incentives that contribute to intervention costs. All cost analyses estimated direct costs related to the implementation of interventions, with substantial variation in terms of cost components. Economic data were most commonly reported for interventions to increase uptake of influenza (*n* = 15) and human papillomavirus (*n* = 11) vaccines. Of the included studies, most related to reminder interventions (*n* = 13).

**Conclusion:**

Our review highlights a lack of both cost data and full economic evaluations for trials of interventions to increase vaccine uptake. Collecting and reporting relevant economic data in a consistent way is vital for enabling informed decision-making around which interventions to implement.

## 1 Introduction

Immunization is a highly cost-effective public health strategy that has contributed to major reductions in hospital admissions, lifelong disability, and deaths from vaccine-preventable diseases [1]. To maximize the effectiveness of immunization programmes, it is essential to have high, timely vaccine coverage, but this is often not achieved [2]. In 2020, the World Health Organization (WHO) published the global strategy to enhance immunization coverage under the Immunization Agenda 2030. To support global strategies for enhancing immunization coverage, we need evidence on which interventions and implementation approaches are most clinically and cost effective [3].

Public health authorities and providers need to balance the necessity of improving vaccine uptake with limited resources and organisational capacity, whilst grappling with competing priorities [4]. While there have been some efforts to report the resources needed to implement strategies to promote vaccine uptake, the associated costs and implications for resources have not been well described [5]. Cost analyses can estimate the additional resources needed to implement an intervention, whilst full economic evaluations can estimate the incremental costs and effects of an intervention compared to alternatives [6]. To enhance efficiency and effectiveness in immunization policy, it is important to understand the costs, as well as the effectiveness, of interventions to promote uptake [7].

While several reviews have examined economic evidence for interventions to promote vaccine uptake (e.g., [8–13]), they have tended to focus on specific interventions or population groups, or are now out of date. A systematic review of economic evidence for 12 interventions recommended by the US Community Preventive Services Task Force found little economic data for interventions targeting uptake of adolescent human papillomavirus vaccination (HPV) and meningitis vaccinations [9]. More recent reviews include Hong et al. (2021) which reviewed costs of interventions that improve children’s vaccine uptake in the United States, and Wang et al. which focused on the use of digital health technology [12]. Although these reviews provide useful information for resource allocation, understanding the costs of interventions, including those for different components would be helpful for vaccine policymakers to inform decisions on the implementation of such interventions [14,15]. Most recently, the UK National Institute for Health and Care Excellence (NICE) conducted a series of systematic reviews of economic evaluations of interventions to improve vaccine uptake (2022) [10,16–22]. In total, they identified 11 studies that conducted cost effectiveness analyses (CEA) or cost utility analyses (CUA), published up to April 2021. The reviews highlighted a lack of interventions targeted at pregnant women and did not include data pertaining to COVID-19 vaccinations. The reviews also highlighted the overall low quality of such analyses and great heterogeneity of costing methods.

This comprehensive review aimed to collate data on costs and economic evaluations of recipient-focused interventions to increase vaccine uptake across a range of populations, settings, and vaccine types, to support resource allocation decisions.

## 2 Methods

This review of economic data was undertaken in parallel to a systematic review on the effectiveness of recipient-focused interventions to increase vaccine uptake [23,24]. Detailed methods for the systematic review are available on PROSPERO [23]. The scope of the systematic review is summarized in Box 1.

## 3 Eligibility criteria, search strategy and study selection

Eligible studies were those reporting economic data (including economic evaluations, cost analyses or intervention costs) pertaining to randomized controlled trials (RCTs) included in the systematic review.

Searches were conducted in two stages. Firstly, to identify RCTs for inclusion in the systematic review a comprehensive search of nine electronic databases (including MEDLINE, Embase, PsycINFO, CINAHL, Web of Science, Cochrane Library and three education databases), clinical trial registrations and thesis repositories was conducted from 2000 to April 2024 [24] (See Box 1). Secondly, targeted searches for economic evidence were conducted in MEDLINE from 2000 to March 2024 and bibliographic records published on the archived NHS Economic Evaluation Database (EED) until 31st March 2015 (see supplementary material for search strategies).

Reflecting the approach to searching, study selection was conducted in two streams. Firstly, during screening and data extraction for the systematic review, which was conducted by two reviewers independently in Covidence [25], any studies reporting relevant cost and economic information were identified [24]. Full text papers were reviewed by one reviewer (YH), and any uncertainties were discussed with a second reviewer (KB). Secondly, the results of the targeted economic searches were screened in Rayaan [26]. Title and abstract screening was conducted by one reviewer (YH), with 20 % double screened (KB) to ensure validity of the process. Full text papers were screened by one reviewer (YH) with 10 % double screened (KB). Any disagreement or uncertainty was discussed with the review team.

## 4 Data extraction and quality assessment

We extracted information on intervention costs and any health economic evaluations of recipient-focused interventions using a standardized Excel data extraction form (YH) with 10 % double-checked (KB). Data items extracted included: total cost of intervention, cost of intervention per person vaccinated, cost per person reached, cost components, quality-adjusted life years (QALYs) and incremental cost-effectiveness ratios (ICERs) from both CUA and CEA. Costs were categorized as operational costs (e.g., staff wages incurred from the intervention implementation or administration fee) and start-up costs (e.g. costs incurred to set up the intervention, such as training or the development of the registry) and indirect (e.g. patient travel costs and costs related to productivity loss costs). Regarding the interventions addressing affordability, incentives were classified into monetary rewards (e.g. offering cash or voucher), lottery entry to win a financial reward, and others. Quality of the economic evidence for each study was assessed by one reviewer (YH) using the 10-item Drummond checklist [6], with 25 % double assessed by a second reviewer (KB).

## 5 Synthesis of findings

Extracted data were tabulated alongside information on intervention ‘reach’, stratified by intervention type and population group. In this review, intervention reach is defined as the number of participants allocated to the intervention arm. Additionally, cost per additional vaccine is defined as the intervention cost per one additional vaccine shot administered in this study.

Data were grouped by intervention type based on the intervention categories used in the systematic review [24] and reported in Box 2. However, for the purposes of this economic review, where studies had multiple ‘active’ intervention arms, interventions were classified according to the arm for which economic data were reported. This means that multi-arm studies could be classified into more than one intervention category if economic data were reported for more than one intervention arm. The only deviation from this approach was that any interventions that included a financial incentive were placed in the ‘Affordability’ category, irrespective of other features of the intervention, since the only economic data reported for these interventions pertained to the cost of the financial incentive.

A narrative synthesis was used due to the expected heterogeneity of the available data [27]. As a result of the variation in currencies used to quantify costs in the economic studies, we converted all monetary values using local inflation rates (adjusted to reflect 2024 prices) and then exchanged to US dollars [28].

## 6 Results

### 6.1 Study selection

**Fig. 1:**
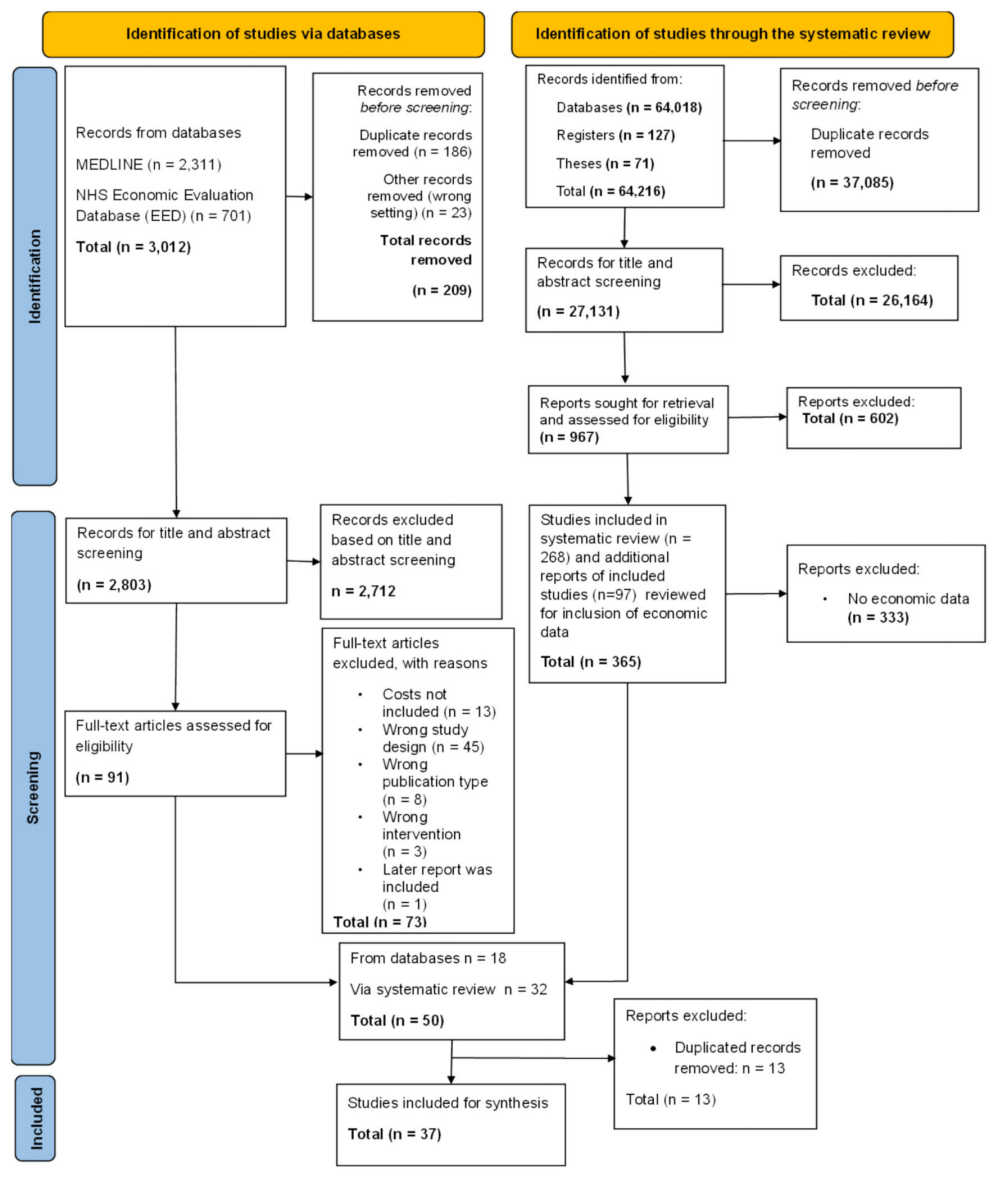
PRISMA flow chart.

We screened 2803 titles and abstracts from the targeted searches for economic data and 365 potentially eligible reports identified from the systematic review. In total, 37 studies were eligible for inclusion in the synthesis. The PRISMA flow chart of the screening process is reported in Fig. 1.

### 6.2 Characteristics of included studies

The majority of studies were conducted in the United States (*n* = 27, 73.0 %) and four were set in the UK. Two studies were conducted in China and the rest were set in Denmark, Sweden, Germany, and Australia. Ten studies were published after 2019.

Most of the studies focused on interventions to increase the uptake of influenza (*n* = 15, 40.5 %) and HPV vaccines (*n* = 11, 29.7 %). Ten other studies targeted at tetanus, diphtheria, and acellular pertussis (Tdap or DTaP). Six studies each addressed vaccines for meningococcus and pneumococcus (*n* = 6, 16.2 %), and five other studies targeted for measles, mumps, and rubella (MMR) vaccines. Three studies focused on interventions for COVID-19 vaccines. The most common target populations for vaccination were adolescents (n = 11, 29.7 %) and adults (*n* = 9, 24.3 %), whilst fewer studies reported on vaccinations for children (*n* = 8, 21.6 %) and older adults (n = 6, 16.2 %).

The majority of interventions were implemented in healthcare settings (*n* = 26, 70.3 %). Six studies were set in educational settings, such as a school or university campus, and four studies were conducted in the community, while one study assessed an online-based intervention.

Sixteen studies (43.2 %) reported full economic evaluations, whilst 10 (27.0 %) conducted cost analyses. The remaining 11 (29.7 %) reported partial intervention costs (financial incentives to encourage vaccine uptake), but did not report either a full economic evaluation or cost analysis.

Among 16 studies reporting full economic evaluations (Table 1), none conducted CUA. One study conducted both CEA and cost benefit analysis (CBA) [29] and the remaining 15 studies reported CEA. Of the 16 studies, five assessed reminder [30–34]; five examined interventions with educational and reminder components [35–39]; three examined educational interventions [29,40,41]; two evaluated the effectiveness of school-located vaccination [42,43]; and one assessed an intervention with multiple components [44]. The characteristics of economic data reported according to intervention type are reported below, followed by findings from full economic evaluations.

### 6.3 Reminder interventions

Among the selected studies, reminder interventions had the most economic data available (*n* = 13 studies). Reminder strategies took different delivery forms such as postcards, letters, text messages, centralized phone calls and home visits.

Eight studies conducted cost analysis and five conducted full economic evaluations. Total costs of the intervention, cost per additional vaccine administered, and cost per person reached are summarized in Table 2. Among the 13 studies evaluating the effectiveness of reminders, nine studies reported cost per person reached and three studies estimated cost per additional vaccine administered.

Cost per person reached ranged between $0.22 and $85 [30,31,33,34,45,48–51] (Table 2). In two cases, cost per person reached tended to be lower when they only estimated personnel expenses or part of intervention costs [49,45]. The costs tended to be higher when more reminders were sent [30,33] or the intervention was implemented in urban areas, often driven by higher wages compared to the rural areas [30,33]. There were substantial variations in the cost per person reached depending on the mode of delivery. The multiple delivery methods tended to lead to higher costs compared to using a single method. For example, the cost per person reached for a combination of different models of delivery (e.g. letter, phone call) ranged between $1.11 - $85.06 per person reached, compared to automated centralized calls ($0.22–53.63 per person reached) [30,31,33,51,48], reminder by mailed letters ($0.66–$2.29 per person reached) [33,51,49,45] or short text messages ($0.30–$14.14 per person reached) [33,51,50].

Only three studies reported a cost per additional vaccine administered [30,33,34] and two others only reported a cost per additional vaccine series completed [31,32] (Table 2). The cost per additional vaccine ranged widely from $20.03 to $1014.94. There was no clear trend between different types of reminders. One study, aiming to increase uptake of vaccines for Tdap, meningococcus or HPV, reported higher cost per additional series completed among the automated phone call arm ($982.47) compared to the mailed reminder arm ($637.58) [32], although a more recent study targeting influenza vaccines reported cost per additional vaccine delivered was higher among the mail arm ($135.20–$1100.41) than the automated call arm ($20.03–$25.04) [33] (Table 1).

Only five studies separately reported the implementation costs for population sub-groups, such as different age groups [34], rural and urban areas [30,33,51], or level of deprivation [32]. One study that targeted a socially deprived population using automated phone calls reported cost averted per person reached per year of $22.92 [32] (Table 1). In another study evaluating reminder letters followed up with a phone call to improve uptake of vaccines for Tdap, or influenza among adult populating aged 19 to 64, and vaccines for Tdap, influenza or pneumococcus among older adults aged 65 and over, they reported different costs per additional vaccine depending on the age groups, indicating the higher costs for a younger and lower risk population ($158.34 per additional vaccine for any target condition) compared to an older and high-risk population target group ($32.57 per additional vaccine for any target condition) [34].

Regarding cost components reported, all eight cost analyses only estimated direct costs involved in the implementation of the strategies for increasing vaccine uptake, and they all separate or did not include the vaccine fee (Table 3). All studies adopted perspectives from local programme providers such as local public health departments, immunization information services and community-based practices (Table 2). However, there was a substantial variation in the components of cost each study reported. Although two studies reported start-up and operational costs [30,36], six other studies only reported personnel and/or material costs that feed into operational costs [13,49,45–48].

### 6.4 Affordability interventions

Eleven studies used a form of financial incentive to encourage vaccine uptake (Table 4). Of these, four evaluated financial incentives to improve uptake of seasonal vaccines for influenza; three evaluated interventions for COVID-19 vaccine uptake; and four assessed interventions for vaccines that entail multiple visits for completion such as HBV and HPV. As we excluded papers reporting provider-focused interventions (see Table 4), all of the financial incentives described are directed at individuals invited for vaccination.

Three types of financial incentives were used: (a) monetary reward, such as providing a cash or voucher upon vaccination (eight studies) [55–58,60,61,63,64]; (b) lottery entry to win a financial reward (three studies) [59,61,62]; and (c) a pay-it-forward scheme (one study) [54] that invites people for vaccination free of charge while inviting participants to donate the cost of other people’s vaccines.

All studies only reported the face value of financial incentives, none conducted cost analyses or full economic evaluations. The value of monetary rewards upon each vaccination received ranged from $1.13 and $40.47. Several studies offered multiple rewards to encourage uptake of multiple doses or complete the series of vaccines (e.g. HBV and HPV vaccines), in which the maximum amount of incentives offered ranged from $57.60 to $92.27.

The value of monetary incentives that participants had a chance to win in a lottery ranged from $5.83 to $5672.65 [59,61,62]. Although two of these had a fixed amount of reward to win and participants entered the lottery upon vaccination [61,59], another study invited participants to draw a lottery to determine the amount of the reward (some people may draw $0) before potential vaccination [62].

### 6.5 Education and reminder interventions

Seven studies assessed the effectiveness of interventions which incorporate both a reminder and an educational component, such as providing a leaflet or decision aid.

Four studies estimated cost per person reached [35,36,38,39], ranging between $0.04 and $555.66. In contrast, five studies reported cost per additional vaccine, which ranged between $1.92 and $700.84 [35–39] (Table 2).

Interventions involving in-person interaction or multiple modes of delivery tend to be more costly. For example, Hambidge et al. (2009) assessed a step-wise reminder for childhood series vaccination delivered through postcard, telephone call, and home visit [38]. Their cost per person reached per month was $37.04, while the cost per additional vaccine was estimated as $422.91. Krieger et al. (2000) evaluated the promotion activities based at community senior centres [37]. The activities consist of diverse programmes such as newsletter article, health fair, posters and mailed reminders. The costs per additional vaccine for pneumonia was $378.09 and $700.84 for influenza.

### 6.6 Education

Three studies evaluated interventions that provided education around vaccination [29,40,41]. All reported full economic evaluations: three CEAs [29,40,41], and one return on investment (ROI) analysis [29] (Table 1).

Two studies assessed educational interventions for influenza [29,40] one for pneumonia vaccines [40] in adults using the perspective of the insurer, and another study evaluated an intervention for MMR [41]. Two studies [29,40] reported partial costs of intervention implementation and estimated the cost of mailing letters as the intervention cost, but it was unclear what each cost exactly entailed. Both recorded the healthcare visits and inpatient admission that related to influenza and/or pneumonia as the outcomes of the interventions.

Berg et al. (2004) calculated ICERs using increase in vaccine uptake as the effectiveness outcome [40]. For the judgement of cost-effectiveness, they concluded that the educational mail was not cost-effective because their estimated CER, $154.39 per additional person vaccinated, was higher than the threshold cost effectiveness ratio (CER) of $46, cited from a previous study [65]. However, incremental savings for the influenza mailing intervention were estimated as $13,286.60 per 10,000 people for Emergency Department visits.

Berg et al. (2008) estimated ROI using the unit cost method and per member per month (PMPM) methodologies [29]. The ROI for the influenza mailing was calculated to be $8.12 and $4.18, respectively, with net saving per person of $18.21 and $23.04, respectively. The authors concluded the mailing intervention was cost-effective for its favourable ROI (Table 1).

One study conducted a CEA of mailing a weblink to a web-based decision aid in comparison with a mailed leaflet or usual care (sending no mails). The costs are estimated separately from the healthcare providers’ perspectives and societal ones, which account for cost and benefits of child or adolescent vaccine recipients and their parents [41]. In their study, parents’ costs of travel to GPs for vaccination were estimated while considering the ease of access to GPs. They estimated that the web-based decision aid had a 72 % probability of being cost-effective based on the healthcare providers’ perspective, in comparison to 22 % chance for the leaflet arm and an 8 % in the usual care arm.

### 6.7 Access interventions

Three studies evaluated school located vaccination programmes. One evaluated school-based vaccination for HPV, meningococcus, and Tdap and reported cost per person reached of $32.95 [53] (Table 2). Two studies conducted cost-effectiveness evaluation and estimated ICERs of school-located vaccination programmes [42,43]. Both adopt a societal perspective that accounts for averted parental costs (e.g., avoiding the travel time required to take a child to a family practice for vaccination). Yoo et al. (2015) explored the difference between the average cost of interventions undertaken in either the first or second year of a school-located influenza vaccination programme in 21 elementary schools [42]. They compared Year 1 and 2 as they anticipated that improved programme delivery and staff efficiency might impact on intervention costs. The mean ICER for each year was estimated in comparison with influenza vaccination in private paediatric practices. They concluded that there was no significant difference in terms of the incremental net cost per vaccinated child accounting for indirect costs compared with influenza vaccine in private paediatric practice in Year 1 ($33.61) and Year 2 ($33.76) (Table 1).

Yoo et al. (2019) conducted a CEA of a school-located immunization programmes at elementary and secondary schools [43]. ICERs of school-located vaccination in elementary schools, secondary schools and all schools were estimated separately from deterministic analysis and probabilistic analysis. The ICERs were decreased for both elementary and secondary schools when taking account of the spillover effects on practices compared to ICERs estimated from the healthcare perspective. The ICERs excluding vaccine purchase for elementary schools and secondary schools are $110.43 and $111.46, respectively, while the ICERs accounting for the spillover impact for elementary schools and secondary schools are $103.76 and $68.80, respectively (Tables 1 and 2).

### 6.8 Multicomponent interventions

Only one study evaluated interventions that consisted of multiple components and reported relevant economic data [44]. This study assessed an intervention consisting of patient tracking of vaccination records, reminder/recall, and home visit by a community vaccine navigator. This study also conducted CEA [44], reporting $677.06 as the cost per additional vaccine and $66.60 as cost per person reached per year (Tables 1 and 2).

### 6.9 Quality assessment of full economic evaluation

Drummond’s 10-item checklist was applied to the 16 studies that reported full economic evaluations [6](Table 5).

Applying the checklist, overall quality of reporting economic evaluation is poor. Even though all 16 studies conducted an incremental analysis of costs and effectiveness, the cost-effectiveness conclusion was rarely discussed. Similarly, how the cost-effectiveness conclusion was reached was often unclear, except in the case of Tubeuf et al. (2014), which used a cost-effectiveness acceptability curve to indicate the likelihood of cost-effectiveness depending on vaccine uptake [41]. In Yoo et al. (2015), ICERs were estimated from the comparison between vaccination based at school-located interventions and private paediatric practices, which lacks a detail of usual care and the comparator [42]. ICERs were also compared across each year of the program (Year 1 and Year 2). In their subsequent study, the ICER of the school-located vaccination intervention was compared to that of a paediatric practice-based vaccination program (versus no vaccination), with estimates of intervention costs drawn from the authors’ earlier work [43]. Furthermore, with four studies that have a time horizon greater than 12 months, none of them discounted costs and consequences [31,36,42,44]. Meanwhile, only two studies reported uncertainty in the estimates of costs and consequences. Yoo et al. (2015; 2019) used modelling to estimate the different uptake and Yoo et al. (2019) conducted a one-way sensitivity analysis for the school-located vaccination in all schools [42,43]. Incremental analysis of costs and consequences was also reported inadequately.

## 7 Discussion

### 7.1 Summary of key findings

We conducted a comprehensive review of economic evidence related to interventions aimed at increasing vaccine uptake in high- and upper middle-income countries. We identified 37 eligible studies reporting economic data on a range of different interventions and vaccine types. Interventions were categorized as reminders, education and reminders, affordability and access; most of the economic data was on reminder interventions. Financial incentives were reported in several RCTs, but no additional costs related the intervention were provided. Sixteen full economic evaluations were identified. Quality assessment found that the reporting was generally weak. Cost per person reached was reported in 15 studies: interventions using reminders (*n* = 10 studies), a combination of reminders and educational components (*n* = 3), multicomponent (n = 1) or interventions to increase access to vaccination (n = 1), ranging from $0.04 to $555.66. However, inconsistent reporting of cost components means that comparing interventions is methodologically challenging.

### 7.2 Interpretation of findings in the context of other evidence

Consistent with previous reviews, we found a lack of cost data and an absence of detailed reporting of cost components. Jacob et al. (2016) covered all population groups but as the review is now somewhat outdated (included studies published to 2012), it is not directly comparable to our review which reflects the contemporary evidence base, including studies on COVID-19 vaccination [9]. Ozawa et al. (2018) included 42 studies in their synthesis. Whilst the number of studies reviewed is similar to our review, we focused on studies from higher income countries [66]. They also report a lack of cost information and that when this was reported it was not broken down into components. Anderson et al. (2018) also noted the paucity of detailed costs, particularly the omission of data pertaining to the cost of illness associated with the relevant vaccine preventable disease [8]. Similarly, costs were predominantly programme costs and interventions were largely deemed to be cost-effective. Ozawa et al. (2018) found that cost data were most commonly reported for reminder interventions [66]. Consistent with our findings, Hong et al. (2021) reported recall and reminder interventions as the least costly per child vaccinated, and similarly multicomponent community-based interventions were most expensive [11].

Although reminders in the form of mailed letters, automated centralized calls and text messages tend to be less costly in terms of cost per person reached, it is inconclusive which approach is more cost effective in terms of cost per additional vaccine received or additional vaccine series completed. Furthermore, caution is needed, as more recent studies have reported an increasing number of autodialled texts and calls being marked as spam, which may negatively impact their effectiveness [36,51]. Although combinations of different types of reminder interventions are common, they tend to involve more resources. Hong et al. (2021) focused only on children, although they also reported recall and reminder interventions as the least costly per vaccination, and similarly multicomponent interventions were most expensive [11]. They highlight that interventions for adolescents were less expensive than those for children aged 0–10.

Despite improvements in cost descriptions over time, variability in how cost components were reported reduced clarity. For example, many studies lacked information on personnel costs or system development expenses. There is growing interest in enhancing the quality of vaccine cost analyses, as evidenced by the publication of several international guidelines in recent years. These include Thinkwell’s *Immunization delivery cost catalogue* [67] and *WHO-led consensus statement on vaccine delivery costing* [68]. While such guidelines primarily target low- and lower middle-income countries, they could still provide valuable direction for implementing cost analyses and economic evaluations of interventions to support vaccine programme implementation in higher income settings. Additionally, cost analyses generally lack estimates of opportunity costs. For decision makers, understanding the cost of averted infection, such as reduced healthcare use or fewer days of sickness leave from work, could be particularly useful for justifying more costly or targeted strategies in the future [69].

Persistent challenges also exist in capturing the full benefit of vaccination [70–72]. Economic modelling can estimate the long-term resource implications and benefits of interventions to increase vaccine uptake beyond the length of a RCT. Models can also be adapted for different vaccines or populations. Although economic models were not included in our synthesis, the data we report could be incorporated into models to inform future decision making. Furthermore, the disaggregated data could be used by decision makers to inform resource allocation decisions, particularly in regard to the costs of incentives. Individuals working in local authorities, for example, do not necessarily have the expertise to interpret complex economic analyses and summarising the data may be more accessible. Indeed, NICE endorse Cost Consequence Analysis (CCA) for the evaluation of public health interventions [73]. The question relating to who may be required to funds an intervention to increase vaccine uptake may also influence the evidence reported. Health system financing is heterogeneous, as is the provision of health care. Full economic evaluations remain limited, and assessments of cost-effectiveness often lack standardization. For example, in the use of cost-effectiveness thresholds or choice and description of the comparators.

### 7.3 Strengths and limitations

Regarding the review process, we conducted a rigorous review utilising a comprehensive two-stage search strategy. Whilst our review inclusion criteria were broad we did restrict to studies from high- and upper-middle income countries. Our findings are therefore not directly transferable to lower-income settings which may differ in terms of several factors that influence resource availability, such as funding and infrastructure. Given these differences, a separate review of economic evidence may be required for lower income settings.

We only included economic data pertaining to RCTs in the underlying systematic review. The systematic review only included RCTs with at least 100 participants randomized meaning that some small trials were excluded, such as Butternheim et al.’s study (2016) evaluating financial incentives and McLaren et al.’s study (2023) assessing the effectiveness of lottery-based incentives [74,75].

There were also some limitations of the available data. For example, there was limited economic evidence available for COVID-19 vaccine uptake interventions, which were rolled out rapidly. There was also a paucity of research examining interventions carried out among older adults or other vulnerable or underserved populations. Most economic evidence pertained to reminder interventions – there was a notable lack of evidence for interventions such as those to improve access to vaccination which may potentially be more costly. The evidence was heterogeneous with regard to cost components and approaches to economic evaluation meaning that statistical synthesis was inappropriate.

## 8 Conclusions

Our review highlights a lack of detailed cost data and a substantial lack of quality of full economic evaluations for interventions to increase vaccine uptake. The majority of economic analyses focus on direct costs, with considerable variation in the components of total costs reported. Future research should collect and report relevant economic data to enable informed decision-making around which interventions to implement to improve vaccine uptake. This is especially important given that all six broad intervention types examined in the systematic review were found to be effective in the network meta-analysis, but decisions are needed on which to prioritize when economic resources are constrained. More comprehensive economic data would be particularly useful for interventions designed to improve access to vaccination, to enhance affordability of vaccination (e.g. financial incentives) and multicomponent interventions, given that these types of interventions appeared most effective in the network meta-analysis [24]. Future research studies should also focus on the transparent reporting of costs by population group or setting to enable decision makers to make equity informed decisions.

## Supplementary Material

**Appendix A.Supplementary Data**

Supplementary data to this article can be found online at https://doi.org/10.1016/j.vaccine.2025.127906.

Search strategies

## Figures and Tables

**Table 1:** Studies reporting full economic evaluations.

Study	Target vaccines	Type of interventions	Perspective	Time horizon	Type of full economic evaluation	Comparator	ICER*^1^ (adjusted)	Economic evaluation conclusion	Other economic measures (adjusted)
Dini 2000 [31]	DTap; Polio; MMR	Reminders	Local health department	34 months	CEA	Control (usual care)	Intervention cost per an additional child completing the series: $258.52 (by 18 months); $442.61 (by 24 months)	Not stated	N/A
Krieger 2000 [37]	Influenza; Pneumococcus	Education & Reminders	Senior centre	6 weeks	CEA	Control (usual care)	Marginal cost per additional vaccine received: $378.09 (Pneumonia vaccine); $700.84 (Influenza)	Not stated	Marginal cost of replicating the intervention: $17,224.12 (with a paid coordinators); $5335.62 (with two volunteers replaced the paid coordinator)
Berg 2004 [40]	Influenza	Education	Health plan (inferred)	5 months	CEA	Control (usual care)	Intervention cost per additional vaccine received: $154.39.	Not cost effective	Benefit cost ratio: $3.68; Net benefits: $80,883.06
Berg 2008 [29]	Influenza	Education	Health plan	5 months	CBA, CEA	Control (usual care)	Not reported	Cost effective	ROI: $8.12 (unit cost); $4.18 (PMPM*^2^). Net saving per person: $18.21 (unit cost); $23.04 (PMPM).
Hambidge 2009 [38]	DTP; Polio, MMR; HBV; Hib; Varicella; Pneumococcus	Education & reminders	Local community centre	12 months	CEA	Control (usual care)	Total intervention cost per additional vaccine received: $422.91	Not stated	N/A
Szilagyi 2011 [44]	Meningococcus; Pertussis; HPV	Multicomponent	Primary care practices	15 months	CEA	Control (usual care)	Intervention cost per additional adolescent fully vaccinated: $677.06	Not stated	N/A
Szilagyi 2013 [32]	Tdap; HPV; Meningococcus	Reminders (mail) Reminders (autodial phone call)	Local health department (inferred)	12 months	CEA	Control (usual care)	Intervention cost per additional adolescent fully vaccinated: $637.58 Intervention cost per additional adolescent fully vaccinated: $982.47	Not stated Not stated	Cost averted per adolescent who received a mailed reminder: $25.81 per year Cost averted per adolescent who received an autodial phone call: $22.92 per year
Tubeuf 2014 [41]	MMR	Education	Healthcare provider Societal	12 months	CEA	Control (usual care), leaflet	N/A	Cost effective (Drawing on a 72 % probability of being cost-effective based on the healthcare providers’ perspective)	Incremental costs versus decision aid (healthcare perspective): $19.50 (usual care); $15.20 (leaflet) Incremental costs versus decision aid (societal perspective): $14.03 (usual care); $18.55 (leaflet)
Yoo 2015 [42]	Influenza	Access	Society	2 years	CEA	Control (practice based vaccination), 1st year and 2nd year of the intervention implementation	Incremental cost per additional vaccine received: $83.78 (Year 1); $84.13 (Year 2); With taking account of indirect costs: $33.61 (Year 1), $33.76 (Year 2)	Not cost effective (no significant difference with control)	N/A
Coley 2018 [35]	HPV	Education & reminders	Local Immunization Information System (inferred)	6 months	CEA	Control (usual care)	Intervention cost for each additional adolescent who started the HPV vaccine series in the six months after receiving a reminder letter: $39.88	Not stated	N/A
Hurley 2018 [34]	Influenza; Tdap (aged 19– 64); Influenza, Tdap, pneumococcus (aged 65 and over)	Reminders (two autodial phone call followed by a postcard)	Local immunization information system	7 months	CEA	Control (usual care)	Intervention cost per additional vaccine received: $158.34 (no high-risk group aged 19–64 years); $79.11 (high-risk group aged 19–64); $32.57 (group aged 65 and over)	Not stated	N/A
Yoo 2019 [43]	Influenza	Access	Society, practice	1 year	CEA	Control (practice based vaccination)	Incremental intervention cost per additional vaccine received (excluding vaccine purchase): $110.43 (elementary school); $111.46 (secondary school);	Not cost effective	N/A
							Incremental intervention cost per additional vaccine received (accounting for observed spillover impact on practice based vaccination): $103.76 (elementary school); $68.80 (secondary school)	Not cost effective	N/A
Kempe 2020 [30]	Influenza	Reminders (up to 1 autodial reminder)	Local immunization information system	4 months	CEA	Control (usual care)	Intervention cost per additional vaccine received: $61.23 (New York)	Cost-effectiveness estimates were higher than those previously reported for	N/A
		Reminders (up to 2 autodial reminders)					Intervention cost per additional vaccine received: $36.99 (Colorado); $80.37 (New York)	the same type of intervention.	N/A
		Reminders (up to 3 autodial reminders)					Intervention cost per additional vaccine received: $89.30 (New York)		N/A
Szilagyi 2020a [36]	HPV	Education & reminders (1 reminder)	Local immunization information system	12 months	CEA	Control (usual care)	Intervention cost per additional vaccine initiation:$2.23 (New York), $1.92 (Colorado); Intervention cost per additional series completion: $2.75 (New York), $2.32 (Colorado)	Not stated	N/A
		Education & reminders (2 reminders)					Intervention cost per additional vaccine initiation: $2.53 (New York), $2.28 (Colorado); Intervention cost per additional series completion: $3.15 (New York), $2.62 (Colorado)		N/A
		Education & reminders (3 reminders)					Intervention cost per additional vaccine initiation:$2.80 (New York), $2.44 (Colorado); Intervention cost per additional series completion: $3.57 (New York), $2.85 (Colorado)		N/A
Szilagyi 2020b [33]	Influenza	Reminders (autodial phone call)	Local immunization information system	3 months	CEA	Control (usual care)	Incremental cost per additional vaccine received: $25.04 (New York); $20.03 (Colorado)	Not stated	N/A
		Reminders (mail)					New York: $1100.41; Colorado: $135.20		N/A
		Reminders (text)					Incremental cost per additional vaccine received: $30.05 (New York)		N/A
Johansen 2023 [39]	Influenza	Education & reminders (*E*-letters with nudge)	Unclear	5 days	CEA	Control (usual care)	Intervention cost per additional vaccine received: $4.75	Not stated	N/A

1*: Incremental Cost Effectiveness Ratio (ICER). 2*: Per member per month (PMPM). CBA: Cost Benefit Analysis. CEA: Cost Effectiveness Analysis.

**Table 2:** Total cost, cost per person reached, cost per additional vaccine among selected studies (excluding ten studies reporting the effectiveness of interventions related to ‘affordability’).

Type of intervention	Study	Country	Vaccine target population	Target vaccines	Reach	Study period	Intervention summary	Perspective	Total cost (adjusted)	Cost per additional vaccine (adjusted)	Cost per reach (adjusted)	Type of economic evaluation
Reminders	Dini 2000 [31]	USA	Children	DTap; Polio; MMR	215 216	34 months	A combination of autodial phone call and letters Letters	Local health department (LHD)	Not reported Not reported	Not reported Not reported	$30.12 Not reported	CEA
					217		Autodial phone call		Not reported	Not reported	Not reported	
Reminders	Terrell-Perica 2001 [45]	USA	Adults	Influenza; Pneumococcus	6528	9 months	Reminder letters	Health plan (inferred)	$4334.16	Not reported	$0.66 per reminder letter	Cost analysis
Reminders	Hull 2002 [46]	UK	Older people	Influenza	605	2 weeks	Telephone appointing in GP	Practice (unclear)	$255.81	Not reported	Not reported	Cost analysis
Education & reminders	Johnson 2003 [47]	USA	Older people (rural areas)	Pneumococcus	10,374	4 weeks (newsprint advertisement) + 6 weeks (TV advertisement)	Advertisement on TV and newspaper + mailed reminder	Unclear	$48,571.79	Not reported	Not reported	Cost analysis
Reminders					10,381 764	Unclear	Mailed reminder Combination (Registry based reminder & recall, outreach activity)		$4603.83	Not reported	Not reported $71.98 – $85.06	
Reminders	LeBaron 2004 [48]	USA	Children	MMR; Hib; Polio; DTaP		22 months		LHD	Not reported	Not reported		Cost analysis
					763		Autodialled				$40.56 – $53.63	
					659		Outreach				$52.29 – 65.36	
Reminders	Kempe 2012 [13]	USA	Adolescents	HPV; Meningococcus; Tdap	133	6 months	Recall up to 2 times by 1 of 3 classroom based reminders	School-based health centre (inferred)	$107.02-$539.73	Not reported	Not reported	Cost analysis
Reminders	Szilagyi 2013 [32]	USA	Adolescents	Tdap; Meningococcus; HPV	1.396	12 months	Mailed reminders Autodialled telephone reminders	LHD (inferred)	$36,029	Not reported	Not reported	CEA
					1.423				$32,619	Not reported	Not reported	
					2072		Mailed recall (at age of 7 months)					
Reminders	Dombkowski 2014 [49]	USA	Children	DTap; Polio; HBV; Pneumococcus; MMR; Varicella	4601	13 months	Mailed recall (at age of 19 months) Mailed reminder (at age of 12 months)	LHD	$45,753.85	Not reported	$1.13 per notification	Cost analysis
					3502							
Reminders	O’Leary 2015 [50]	USA	Adolescents	Tdap; Meningococcus; HPV	2228	12 months	Reminder/recall by a short messaging service	Practice (healthcare provider)	$1153-4578 per practice	Not reported group aged 19–64); $32.57 (group aged 65 and over)	$3.56 – $14.14	Cost analysis
Reminders	Hurley 2018 [34]	USA	Adults	Influenza; Tdap (aged 19–64); Influenza, Tdap, pneumococcus (aged 65 and over)	17,951	7 months	Two autodial phone call followed by a postcard	Local immunization information system	$19,782.99		$1.11	CEA
					New York: 29,003; Colorado: 7910		Centralized reminder/recall system: Autodialled	Local immunization information system	New York: $6542; Colorado: $4854	New York: $25.04; Colorado: $20.03	New York: $0.35; Colorado: $0.61	
Reminders	Szilagyi 2020b [33]	USA	Adolescents	Influenza	New York: 4779; Colorado: 8007	3 months	Centralized reminder/recall system: Mail		New York: $10,513.38; Colorado: $17,277.35	New York: $1100.41; Colorado: $135.20	New York: $2.20; Colorado: $2.15	CEA
					New York: 29,090		Centralized reminder/recall system: Text		New York: $ 5768.71	New York: $30.05	New York: $0.30	
					30,087		Up to 1 auto-dial		Colorado: $2990.19;	New York:	Colorado: $0.22; New Yor: $0.29	CEA
							reminders		New York: $4885.84	$61.23		
Reminders	Kempe 2020 [30]	USA	Children	Influenza	30,031	4 months	Up to 2 auto-dial reminders	Local immunization information system	Colorado: $3843.61; New York: $6593.97	Colorado: $36.99; New York: $80.37	Colorado: $0.28; NY: $0.40	
					30,017		Up to 3 auto-dial reminders		Colorado: $4629.43; New York: $8318.68	New York: $89.30	Colorado: $0.34; New York: $0.51	
Reminders	Gurfinkel 2021 [51]	USA	Adolescents	HPV	Colorado: 6795, New York: 9911 New York:	25 months	Centralized autodialled call Centralized SMS	Local immunization information	Colorado: $4653.28; New York: $4282.72 New York:	Not reported	Colorado: $0.34; New York: $0.44 New York:	Cost analysis
					9910		text	system	$4170.05		$0.35	
					Colorado: 6860		Centralized mail		Colorado: $24,683.53	Pneumonia	Colorado: $2.29	
Education & reminders	Krieger 2000 [37]	USA	Older people	Influenza; Pneumococcus	622	6 weeks	Immunization promotion activities	Local senior centre	$26,016	vaccine: $378.09; Influenza: $700.84	Not reported	CEA
							Stepped					
Education & reminders	Hambidge 2009 [38]	USA	Children	DTaP; Polio; MMR; Hib; HBV; Varicella; Pneumococcus	409	12 months	reminder intervention (postcard, telephone reminder, then home visit)	Local community health centre	$226,710.20	$422.91	$555.66 (total); $37.04 (monthly)	CEA
Education & reminders	Suh 2012 [52]	USA	Adolescents	Tdap; Meningococcus; HPV (female)	800	19 months	Up to 2 letters and 2 autodialled telephone calls	Local immunization information system	$3855.37-$4906.69	Not reported	Not reported	Cost analysis
Education & reminders	Coley 2018 [35]	USA	Adolescents	HPV	81,558	6 months	Reminder & educational letters	Local immunization information system (inferred)	$74,718.99	$39.88	$0.88 per reminder	CEA	
					15,546		Autodialled phone reminders (1 reminder)			Per vaccine initiation: $2.23 (New York), $1.92 (Colorado); Per vaccine	New York: $0.83; Colorado: $0.64		

Education & reminders	Szilagyi 2020a [36]	USA	Adolescents	HPV	15,501	12 months	Autodialled phone reminders (2 reminders)	Local immunization system	Not reported	initiation: $2.53 (New York), $2.28 (Colorado); Per vaccine	New York: $0.94; Colorado: $0.71	CEA	

					15,469		Autodialled phone reminders (3 reminders)			initiation: $2.80 (New York), $2.44 (Colorado)	New York: $1.04; Colorado: $0.79		
Education & reminders	Johansen 2023 [39]	Denmark	Older people	Influenza	481,965	5 days	Electronic letters with different types of nudge Tiered reminder/recalls including immunization	Unclear	Not reported	$4.75	$0.04 per letter	CEA	

Multicomponent	Szilagyi 2011 [44]	USA	Adolescents	Meningococcus; Pertussis; HPV	3839	15 months	tracking, telephone or mailed reminder/recall, and home visits	Primary care practices	Not reported	$677.06	$66.60per year	CEA	
Access	Daley 2014 [53]	USA	Adolescents, 6th-8th grade	HPV; Meningococcal; Tdap	3144	1 year	School-located vaccination & insurance billing	Community vaccinator	Not reported	Not reported	$32.95	Cost analysis	
Access	Yoo 2015 [42]	USA	Children, Year 1 & 2	Influenza	9027 (Year 1); 9145 (Year 2)	2 years	School-located vaccination	Society	Not reported	With taking account of indirect costs: $33.61(Year 1), $33.76 (Year 2) $110.43 (elementary school); $111.46	Not reported	CEA	
Access	Yoo 2019 [43]	USA	Children	Influenza	21,696 (elementary school); 9488 (secondary school)	1 year	School-located vaccination	Society, practice	Not reported	(secondary school); Accounting for spillover impact on practice: $103.76 (elementary school); $68.80 (secondary school)	$3.48 (School cost +program cost; all school)	CEA	
Education	Berg 2004 [40]	USA	All age	Influenza	82,364	5 months	Direct mailing	Health plan (inferred)	$66,590	$154.39	Not reported	CEA
Education	Berg 2008 [29]	USA	Older adults	Influenza	26,474	5 months	Direct mailing	Health plan	$53,272.78	Not reported	Not reported	CBA, CEA
Education	Tubeuf 2014 [41]	UK	Children	MMR	85 48	12 months*^1^	Decision aids Leaflet	Society, healthcare provider (NHS)	Not reported	Not reported Not reported	Not reported Not reported	CEA

*1: During the 17 months of recruitment and follow-up, all cost expenditures were assumed to occur within 12 months from initial contact. CBA: Cost Benefit Analysis. CEA: Cost Effectiveness Analysis. DTap/Tdap: Diphtheria, Tetanus, acellular Pertussis. MMR: Measles, Mumps, Rubella. Hib: *Haemophilus influenzae* type b. HBV: Hepatitis B Virus. HPV: Human Papilloma Virus.

**Table 3:** Cost components reported in cost analyses.

Study	Country	Target vaccination and population	Type of intervention	Perspective	Type of direct cost(s)	Cost component(s)
Terrell-Perica 2001 [45]	USA	Influenza and pneumococcal vaccine for adults	Reminders	Health plan (inferred)	Operational cost only	Private contractor cost for labour; materials and mailing a reminder letter
Hull 2002 [46]	UK	Influenza for older adults	Reminders	Practice (inferred)	Operational cost	Personnel cost for operating the intervention
Johnson 2003 [47]	USA	Pneumococcal vaccine for older adults	Reminders	Unclear	Operational cost only	No details provided
LeBaron 2004 [48]	USA	MMR, Hib, polio, and DTaP vaccines for children	Reminders	Local health department (LHD)	Operational cost only	Registry cost, direct annual cost of maintaining the registry, cost to each provider of participating in the registry
Hambidge 2009 [38]	USA	DTaP, polio, MMR, Hib, HBV, varicella, and Pneumococcal vaccines for children	Education & Reminders	Programme provider (inferred)	Start-up cost and operational cost	All personnel, mailings, telephone calls, home visits, and creation of the reminder/recall database
Kempe 2012 [13]	USA	HPV, Tdap and meningococcal vaccines for adolescent	Reminders	School-based health centre (inferred)	Operational cost	Personnel and supply costs
Suh 2012 [52]	USA	Tdap, HPV and meningococcal vaccines for female adolescents	Education & Reminders	Paediatric practices	Start-up cost and operating cost	1-time cost that would be incurred in developing recall/reminder system and operating personnel and supply costs
Dombkowski 2014 [49]	USA	Hib vaccine for children	Reminders	LHD	Operational cost only	Personnel cost for operation
Daley 2014 [53]	USA	HPV vaccine for adolescents	Access	Community vaccinator	Operational cost only	Administrative cost
O’Leary 2015 [50]	USA	Tdap, HPV, influenza and meningococcal vaccines for adolescents	Reminders	Practices	Start-up cost and operating cost	Development of the text message, staff training, data management, and staff training, data management, and implementation
Gurfinkel 2021 [51]	USA	HPV vaccine for adolescents	Reminders	Local immunization information system	Start-up cost and operational cost	Consensus building and preliminary work; training; software; collaboration; implementation meetings; and reminders
Johansen 2023 [39]	Denmark	Influenza vaccine for older adults	Education & reminders	Unclear	Operational cost only	Mailing cost

DTap/Tdap: Diphtheria, Tetanus, acellular Pertussis. MMR: Measles, Mumps, Rubella. Hib: *Haemophilus influenzae* type b. HBV: Hepatitis B Virus. HPV: Human Papilloma Virus.

**Table 4:** Studies evaluating the effectiveness of financial incentives.

Study	Country	Target vaccination and population	The value of incentive (adjusted)	Type of interventions and summary of financial incentive
Qin 2023 [54]	China	HPV vaccine for female adolescent (15–18 yrs)	A subsidy of $ 158.80 per person	Affordability (Pay-it-forward): Informing the market prices of HPV vaccines and that previous participants donated to support participant’s vaccination
Topp 2013 [55]	Australia	HBV vaccine for adults (age 16 and over)	$29.89	Affordability (monetary reward): Cash reward for each vaccination (2 times)
Weaver 2014 [56]	UK	HBV vaccine for adults (18–65 yrs)	Fixed rate of $19.20 or escalating late from $9.60 to $28.80	Affordability (monetary reward): Fixed value contingency management (three $19.20 vouchers), or escalating value contingency management ($9.60, $19.20, and $28.80 vouchers)
Mantzari 2015 [57]	UK	HPV vaccine for female adolescents (16-18 yrs)	$92.27	Affordability (monetary reward): Voucher for completing 3 vaccines
Campos-Mercade 2021 [58]	Sweden	COVID-19 vaccine for adults (18-49 yrs)	$30.03	Affordability (monetary reward): Monetary incentive if they got vaccinated within 30 days of the vaccine
Baskin 2018 [59]	USA	Influenza vaccine for adults (age 18 and over)	Chance to win a $ 134.89 voucher	Reminders, Affordability (lottery-based reward): Campus-based intervention and chance to win a voucher in one in three
Bronchetti 2015 [60]	USA	Influenza vaccine for adults (age 18 and over)	$40.47	Education & reminder, Affordability (monetary reward), Multiple components: campus-based and cash upon vaccine uptake
NCT05012163 [61]	USA	Influenza vaccine for adults (age 18 and over)	Chance to win up to $5672.65	Affordability (monetary reward; lottery-based award), Reminders: a $1.13 scratch-off lottery ticket (with top prize of $5000)
			$1.13 in cash	Affordability (monetary reward; lottery-based award), Reminders: $1.13 in cash
Shen 2024 [62]	China	Influenza vaccine for older adults	Chance to win $5.83, $11.65, or $17.48	Affordability (Lottery-based reward): participants are invited to a raffle to determine the value of financial incentives from $0 to $17.48
NCT05534061 2022 [63]	USA	COVID-19 vaccine for adults (age 18 and over)	$10.23	Affordability (monetary reward), Reminders, Multiple components: $10.23 financial incentive for vaccination and $10.23 financial incentive for testing plus a brief feedback-based motivational enhancement intervention. Affordability (monetary reward): $10.23 financial incentive for vaccination and $10.23 financial incentive for testing Affordability
Ternovski 2024 [64]	Germany	COVID-19 vaccine for adults (over 18 yrs)	$29.66 - $59.31	(monetary reward): receive up to $59.31 upon completion

**Table 5:** 10-Item Drummond Checklist for the 16 economic evaluations.

	Was a well-defined question posed in an answerable form?	Was a comprehensive description of the competing alternatives given?	Was there evidence that the programme’s effectiveness was established?	Were all the important and relevant costs and consequences for each alternative identified?	Were costs and consequences measured accurately in appropriate physical units?	Were costs and consequences valued credibly?	Were costs and consequences adjusted for differential timing?	Was an incremental analysis of costs and consequences of alternatives performed?	Was uncertainty in the estimates of costs and consequences adequately characterized	Did the presentation and discussion of study results include all issues of concern to users?
Dini 2000 [31]	No	Yes	Yes	Yes	No	No	No	Yes	No	No
Krieger 2000 [37]	Yes	No	Yes	No	No	No	N/A	Yes	No	No
Berg 2004 [40]	Yes	Yes	Yes	No	No	No	N/A	Yes	No	No
Berg 2008 [29]	Yes	No	Yes	No	Yes	No	N/A	No	No	No
Hambidge 2009 [38]	Yes	No	Yes	No	No	No	N/A	Yes	No	No
Szilagyi 2011 [44]	Yes	Yes	Yes	Yes	No	No	No	Yes	No	No
Szilagyi 2013 [32]	Yes	Yes	Yes	Yes	Yes	Yes	N/A	Yes	No	No
Tubeuf 2014 [41]	Yes	Yes	Yes	Yes	Yes	Yes	N/A	Yes	No	Yes
Yoo 2015 [42]	Yes	No	Yes	Yes	Yes	Yes	No	Yes	Yes	Yes
Coley 2018 [35]	Yes	No	Yes	No	No	No	N/A	Yes	No	No
Hurley 2018 [34]	Yes	No	Yes	Yes	No	No	N/A	Yes	No	No
Yoo 2019 [43]	Yes	No	Yes	Yes	Yes	Yes	N/A	Yes	Yes	Yes
Kempe 2020 [30]	Yes	No	Yes	Yes	No	No	N/A	Yes	No	No
Szilagyi 2020a [36]	Yes	Yes	Yes	Yes	Yes	No	No	Yes	No	No
Szilagyi 2020b [33]	Yes	Yes	Yes	Yes	Yes	No	N/A	Yes	No	No
Johansen 2023 [39]	Yes	No	No	No	No	No	N/A	Yes	No	No

## Data Availability

This is a secondary analysis of published studies.

## Box 1 Scope of the systematic review of interventions to increase vaccine uptake.

**Table:**

Domain	Eligibility criteria
Type of vaccines	All universal and selective/targeted vaccinations on the UK immunization schedule. Studies on all population groups living in the community and eligible for vaccination (or carers of those eligible for vaccination), including parents of young children, adolescents and adults. Studies on specific clinical risk groups were excluded. We excluded studies targeting hospital inpatients, prisoners, and residents of care/nursing homes. We excluded studies focussed on healthcare workers. Any type of intervention targeting the intended recipients of vaccines or their caregivers to increase demand for, or access to, vaccination. We excluded interventions aimed at the provider or healthcare system (e.g. provider training or provider incentives).
Participants	
Interventions	Interventions aimed at both the intended vaccine recipients and providers or healthcare systems were excluded, unless effectiveness data was available for the component targeting the intended recipients of vaccines alone.
Outcome of interest	Vaccine uptake, including single vaccinations and/or completion of a full vaccination course.
Study design	Randomized controlled trials (RCTs; cluster or individually randomized) with more than 100 participants randomized. Cluster RCTs must have at least three intervention sites and three control sites.
Publication period and setting	Studies published from 2000 onwards, undertaken in high and upper-middle income countries as defined by the World Bank in July 2022.

## Box 2 Category of recipient-focused interventions to increase vaccine uptake.

**Table:**

Intervention	Definition used to categorize interventions in this review
Access	Interventions aimed at increasing access to vaccinations. Access can refer to vaccines being provided at different locations, different times (e.g. weekends), extended times (e.g. weekends and early morning), and opportunistic vaccinations.
Education	Interventions that aim to increase awareness and understanding about the vaccine/s. In this review education will refer to interventions that provide information and education. This may include providing information about the recommended schedule, vaccine safety, efficacy, perceived severity and risk associated with the immunization preventable disease.
Reminders	Interventions that aim to increase vaccine uptake via the provision of reminders. We will use reminders to include both an initial invitation to be vaccinated when a routine vaccination becomes due as well as any additional reminders/recall before the vaccine is due or once it becomes overdue.
Affordability	Interventions focused on increasing the affordability of vaccination include those that aim to increase uptake by providing financial incentives as well as those that provide free vaccines or payments to cover financial or time costs.
Education and reminders*	Any intervention that provides information about the vaccine and also has a reminder component.
Multicomponent	Where studies have features of two of the above categories, we assessed which was the most prominent feature by assessing which feature the author highlighted in the title or methods of the study and assessing the content of the interventions). Where a prominent feature could be established, we categorized the study as such. Where interventions included two categories, but there was not an obvious prominent category, rather the categories appeared equally important, we categorized these as multicomponent.
Control	Includes standard care (where participants in the control group continue to receive standard vaccination practices), no intervention, and attention control (where participants in the control group receive an intervention similar in design to the active intervention group, but where the content is not vaccine-based e.g. general health education provided rather than vaccine education)

^*^ These interventions were grouped since they are often delivered together. The separate ‘Education’ and ‘Reminders’ categories were used strictly for interventions that included only those individual strategies. The ‘Multicomponent’ category was intended to be used sparingly, as findings from this category are more difficult to interpret given the mixture of intervention strategies included.

## Abbreviations

CBA: Cost Benefit Analysis
CEA: Cost Effectiveness Analysis
CUA: Cost Utility Analysis
DTap/Tdap: Diphtheria, Tetanus, acellular Pertussis
Hib: *Haemophilus influenzae* type b
HBV: Hepatitis B Virus
HPV: Human Papilloma Virus
ICER: Incremental Cost-Effectiveness Ratio
MMR: Measles, Mumps, Rubella
QALYs: Quality-Adjusted Life Years
RCT: Randomized Controlled Trial
ROI: Return On Investment
