# Supplementary material for: A synthesis of economic data from randomized trials of recipient-focused interventions to increase vaccine uptake
Source: Vaccine. Author manuscript; Available in PMC 2025 Nov 25. (PMC7618402; doi:10.1016/j.vaccine.2025.127906)
Supplement: Search strategies [file EMS210396-supplement-Search_strategies.docx]

**Search strategies**

1. **Search strategy for systematic review component network meta-analysis**

**Ovid MEDLINE(R) ALL** <1946 to April 12, 2024>

Update – RCTs – All Interventions

[*Vaccination*]

1 exp Vaccination/ 113921

2 immunization/ 54143

3 immunization schedule/ 11551

4 Immunization programs/ 13050

5 (immuni#ation? or vaccination? or revaccination? or reimmuni#ation?).tw,kf. 295082

6 (vaccin* adj (uptake? or coverage)).tw,kf. 18822

7 or/1-6 349611

[*Access Interventions*]

8 (*Vaccination/ or *Immunization/) and "Organization & Administration".fs. 1551

9 *Preventive Health Services/ 9142

10 Health Services Accessibility/ 86791

11 ("after hours" or "out of hours" or (extend* adj2 hours)).tw,kf. 4792

12 ((extend* or weekend? or early or earlier or late or later or evening? or commuter?) adj4 (clinic? or service? or appointment? or session?)).tw,kf. 13860

13 Mobile Health Units/ 3943

14 ((mobile adj4 (clinic? or health unit? or health care or healthcare or health van? or hospital? or site or sites)) or field hospital?).tw,kf. 4940

15 (temporary adj4 (centre? or center? or clinic? or facility or facilities or health care or healthcare or health unit? or service? or site or sites)).tw,kf. 979

16 (walkin or "walk in" or popup or pop-up or popin or "pop in" or "pop by" or "drop by").tw,kf. 5196

17 ((dropin or "drop in") adj4 (centre? or center? or clinic? or facility or facilities or health unit? or service? or site or sites)).tw,kf. 748

18 (drive-in or drive-through).tw,kf. 2710

19 (supermarket? or grocery store? or drug store? or instore? or in-store? or shopping cent* or retail unit? or mall or malls or out-of-town).tw,kf. 12959

20 (sporting venue? or stadium? or stadia or arenas).tw,kf. 4944

21 ((urban or rural) adj4 (access* or availability)).tw,kf. 4404

22 (escort* or group visit*).tw,kf. 2260

23 (health* adj (visit* or supervis*)).tw,kf. 6301

24 Safety-net Providers/ 1391

25 ((safety-net or safetynet) adj3 (clinic? or hospital? or provider? or system? or health care or healthcare)).tw,kf. 3581

26 exp Community Participation/ 48588

27 Community Health Centers/ or Community Health Services/ or exp Community Health Nursing/ or Community Mental Health Services/ or Community Networks/ 84585

28 Public Health Nursing/ 10260

29 ((community or public or civic or communal or municipal) adj4 (facility or facilities or provider? or setting? or venue* or located or location? or building* or regional* or clinic? or hall or halls or centre* or center* or space or spaces or site or sites)).tw,kf. 105266

30 (communit* adj4 (advoc* or awareness or champion* or compliance or educat* or engag* or involv* or participat* or uptake?)).tw,kf. 56020

31 (community group? or (communit* adj4 (activ* or campaign* or implement* or intervention or policy or policies or program* or pilot or strategy or strategies or workshop?))).tw,kf. 67151

32 ((community or community health*) adj (care or unit? or nurs* or service? or volunteer*)).tw,kf. 16300

33 (community adj4 (dwelling or based or delivered or located or location? or led or run or set or setting?)).tw,kf. 149067

34 (communit* adj4 (rural or urban or inner city)).tw,kf. 30674

35 Substance Abuse Treatment Centers/ 5427

36 ((drug? or substance?) adj4 (abuse or treat* or rehab*) adj4 (centre? or center? or clinic? or facility or facilities or health care or healthcare or health unit? or service? or site or sites)).tw,kf. 6298

37 ((family or families or carer? or parent* or office or work*) adj (based or delivered)).tw,kf. 23284

38 Community Pharmacy Services/ or Pharmacy/ 16242

39 (pharmacy or pharmacies or pharmacist?).tw,kf. 91295

40 *General Practice/ or *Family Practice/ 52346

41 (((general practi* or family practi* or primary care) adj7 (intervention? or program* or project? or service? or study or trial)) and (access* or uptake? or cover* or rate or rates or visit*)).tw,kf. 23627

42 Family Nursing/ or Maternal-Child Nursing/ 3512

43 Prenatal Care/ or Perinatal care/ or Maternal Child Health centers/ 40181

44 Peripartum Period/ or Postpartum Period/ 33712

45 ((prenatal or pre-natal or antenatal or ante-natal or pregnan* or perinatal or peri-natal or postpartum or post-partum) adj4 (care or centre? or center? or clinic? or facility or facilities or health care or healthcare or health unit? or service? or site or sites)).tw,kf. 60062

46 (mother? adj4 (baby or babies) adj4 (care or centre? or center? or clinic? or facility or facilities or health care or healthcare or health unit? or service? or site or sites)).tw,kf. 613

47 ((midwif* or widwiv*) adj4 (based or delivered or led or run or care or centre? or center? or clinic? or facility or facilities or health care or healthcare or health unit? or service? or site or sites)).tw,kf. 4021

48 (mother? adj4 program*).tw,kf. 2264

49 (well-child adj (care or clinic)).tw,kf. 794

50 ((women adj2 Infants adj2 children) or (WIC adj3 (program* or service? or support*))).tw,kf. 1731

51 exp Child Day Care Centers/ 6326

52 ((child or children* or day or daycare) adj4 (centre? or center?)).tw,kf. 19604

53 Schools, Nursery/ 1512

54 ((nursery or nurseries or kindergarten* or preschool* or pre-school*) adj4 (based or delivered or located or location? or led or run or set or setting?)).tw,kf. 2247

55 ((nursery or nurseries or kindergarten* or preschool* or pre-school*) adj4 (clinic or clinics or health* or nurs* or program* or vaccin* or immuni#ation?)).tw,kf. 17683

56 ((nursery or nurseries or kindergarten* or preschool* or pre-school*) adj7 (accessibility or "access to" or uptake? or cover* or rate or rates)).tw,kf. 1551

57 (play school* or play group?).tw,kf. 179

58 School Health Services/ 18419

59 Schools/ and (exp vaccines/ or Vaccination/) 978

60 ((school? or highschool?) adj4 (based or delivered or located or location? or led or run or set or setting?)).tw,kf. 34598

61 (school? adj4 (clinic? or health care or healthcare or health unit? or nurs* or vaccin* or immuni#ation?)).tw,kf. 22557

62 (school? adj7 (accessibility or "access to" or uptake? or cover* or rate or rates)).tw,kf. 9776

63 ((universit* or colleg*) adj4 (based or delivered or located or location? or led or run or set or setting? or students)).tw,kf. 107150

64 ((universit* or colleg*) adj4 (clinic? or health care or healthcare or health unit? or nurs* or program* or vaccin* or immuni#ation?)).tw,kf. 46716

65 (library adj4 (based or delivered or located or location? or led or run or set or setting?)).tw,kf. 5011

66 (public library or (library adj4 (clinic? or health care or healthcare or health unit? or nurs* or program* or based or delivered or setting? or vaccin* or immuni#ation?))).tw,kf. 5710

67 ((leisure or recreation or sports) adj4 (centre? or center? or facility or facilities)).tw,kf. 1947

68 (sports hall? or gym or gyms or gymnasium?).tw,kf. 2812

69 (faith adj2 organi#ation?).tw,kf. 619

70 ((religion or religious) adj4 (building? or centre? or center? or facility or facilities or delivered or located or location? or led or run or set or setting?)).tw,kf. 705

71 ((faith or church* or mosque? or synagogue?) adj4 (clinic? or health care or healthcare or nurs* or program* or based or delivered or setting? or vaccin* or immunis* or immuniz*)).tw,kf. 3355

72 Transcultural Nursing/ 3466

73 "Religion and Medicine"/ 11672

74 home care services/ or home health nursing/ or home nursing/ 44595

75 House Calls/ 4224

76 (housecall? or house call? or outreach or out-reach or (door adj2 door) or ((home or homes) adj4 visit*) or (home adj4 (immuniz* or immunis* or vaccinat*))).tw,kf. 56508

77 adult day care centers/ or Senior Centers/ 255

78 Homes for the Aged/ or Housing for the Elderly/ 16383

79 assisted living facilities/ or group homes/ or exp nursing homes/ 47346

80 residential facilities/ 5761

81 (assisted living or ((residential or nursing) adj4 home*)).tw,kf. 46448

82 (care home? or rest home? or long* term care facilit*).tw,kf. 13322

83 orphanages/ or Foster Home Care/ 4366

84 (orphange? or foster care or foster home* or kinship care or children* home*).tw,kf. 4399

85 exp Education, Special/ 15644

86 (special adj4 (education or school*)).tw,kf. 7131

87 halfway houses/ 1071

88 exp Homeless Persons/ 11428

89 (homeless* or home less* or hostel? or ((halfway or half-way) adj (house? or home?))).tw,kf. 15871

90 Prisons/ 11486

91 (prison? or ((custody or correctional or detention* or reformato* or remand) adj4 (centre* or center* or facilit* or institut*))).tw,kf. 17275

92 Ambulatory Care Facilities/ 22899

93 Emergency Service, Hospital/ 89858

94 Outpatient Clinics, Hospital/ 15853

95 ((outpatient? or out-patient? or out-of-hospital) adj7 (intervention? or program* or project? or service? or study or trial)).tw,kf. 47402

96 ((outpatient? or out-patient?) adj4 (centre? or center? or clinic? or unit? or visit*)).tw,kf. 72397

97 (ambulatory care or ambulance? or paramed* or para-med* or paraprofessional? or para-professional? or emergency department? or (accident* adj2 emergenc*) or ED visit*).tw,kf. 173176

98 (ED immunis* or ED immuniz* or ED vaccinat*).tw,kf. 20

99 (case management or case manager? or case worker?).mp. 21135

100 (interpreter* or translator*).tw,kf. 6938

101 (social adj (support or work*)).mp. 140996

102 Vaccination/nu or vaccin* manager?.tw,kf. 174

103 Occupational Health/ 36885

104 ((work adj2 (based or delivered or place? or site or sites)) or workbased or workplace? or worksite?).tw,kf. 73718

105 ((vaccinat* or immuni#ation? or reimmuni#ation?) adj4 (occupational health or employee? or employer? or frontline or front-line or personnel or provider? or staff or worker? or work force or workforce or "at work")).tw,kf. 5886

106 (opportun* adj (vaccination? or immuni#ation?)).tw,kf. 60

107 (opportun* adj4 (catch-up or catchup or boost* or revaccinat* or reimmunis* or reimmuniz* or re-vaccinati* or re-immunis* or re-immuniz*)).tw,kf. 193

108 or/8-107 1767514

109 7 and 108 36055

[*Reminder Interventions*]

110 Reminder Systems/ 3818

111 ((remind* or recall* or messag* or alert*) adj4 system?).tw,kf. 6606

112 (reminder? or prompts or prompting or prompted or nudge?).tw,kf. 89967

113 ((immuni* or vaccin*) adj4 (recall* or remind*)).tw,kf. 1261

114 (recall* adj4 (alert* or appointment? or based or boost* or calendar or campaign* or central* or correspond* or initiative? or intervention? or invit* or letter? or mail or email or message? or notice? or phone? or postal or "by post" or program* or prompt* or regist* or remind*)).tw,kf. 4485

115 (remind* adj4 (alert* or appointment? or based or boost* or calendar or campaign* or central* or correspond* or initiative? or intervention? or invit* or letter? or mail or email or message? or notice? or phone? or postal or "by post" or program* or prompt* or regist* or recall*)).tw,kf. 5185

116 ((recall* or remind*) adj4 (app* or auto* or computer* or digital* or electronic or tele* or SMS or text*)).tw,kf. 7335

117 ((recall* or remind*) adj4 (adults or patients or individuals or parent* or carer? or mothers or fathers or family or families or men or women)).tw,kf. 9106

118 ((recall* or remind*) adj4 (employee? or employer? or frontline or front-line or personnel or provider? or staff or worker? or work force or workforce)).tw,kf. 1097

119 (remind* or recall*).ti. 13432

120 ((auto* or computer*) adj4 (alert* or messag*)).tw,kf. 2378

121 (autodial* or (auto* adj4 (call* or dial* or tele*))).tw,kf. 6505

122 correspondence as topic/ 2331

123 postal service/ 2443

124 letter/ 1248841

125 (letter? or correspondence).tw,kf. 160105

126 invitation?.tw,kf. 8068

127 ((immuni* or vaccin*) adj4 letter?).tw,kf. 301

128 (communication? and (vaccin* or Immuni#ation*)).ti,kf,hw. 2880

129 (mail* out? or circulars).tw,kf. 779

130 electronic mail/ or text messaging/ 7513

131 posters as topic/ or poster?.tw,kf. 12479

132 pamphlets/ or (pamphlet? or leaflet? or brochure?).tw,kf. 34588

133 (recei* adj4 (vaccin* or immuni#ation?) adj4 (alert* or information or messag*)).tw,kf. 307

134 decision support system?.mp. 16605

135 (decision adj4 making adj4 (auto* or system? or technique?)).tw,kf. 5671

136 ((activat* or auto* or check* or review or update or up-to-date) adj3 (health* or medical or patient?) adj3 record?).tw,kf. 18860

137 (immunosurveillance or immuno-surveillance).mp. 3949

138 (postcard* or post* card*).tw,kf. 12302

139 or/110-138 1611825

140 7 and 139 18015

141 (vaccin* adj4 (text? or tele* or recall* or remind* or prompt* or postcard* or post* card* or postal or nudg* or messag* or mail or email? or letter? or invit* or autodial* or auto* dial* or alert*)).tw,kf. 3013

142 Reminder Systems/ and (Primary Prevention/ or Preventive Health Services/) 118

143 140 or 141 or 142 19495

[*Educational Interventions*]

144 HEALTH PROMOTION/ or (public health adj (campaign? or message?)).mp. 84772

145 Health Education/ 64190

146 (health adj (education or promotion)).tw,kf. 86325

147 Knowledge/ or Patient Medication Knowledge/ 15327

148 Health Knowledge, Attitudes, Practice/ and (uptake? or coverage).mp. 6475

149 Patient Acceptance of Health Care/ and (uptake? or coverage).mp. 6262

150 Education/ 21535

151 Health Literacy/ 9858

152 Patient Education as Topic/ 88464

153 consumer health information/ 4336

154 National Health Programs/ 33710

155 Government Publications as Topic/ 698

156 Health Fairs/ or health fair?.tw,kf. 1026

157 (education* adj4 (campaign? or information* or intervention? or initiative? or message? or program* or promotion* or strateg* or tailor* or tool* or target* or study or trial)).tw,kf. 153771

158 curriculum/ or curricul*.tw,kf. 128862

159 (literacy or illiterat*).tw,kf. 37992

160 (campaign or education*).ti. 192646

161 ((client? or consumer? or patient? or public) adj4 (educat* or teach* or train*)).tw,kf. 120780

162 (educat* and inform*).ti,kf,hw. or "educat* and inform*".ab. 33558

163 (fact sheet? or factual information or informational).tw,kf. 13211

164 Information Seeking Behavior/ 3292

165 Information Dissemination/ 19559

166 persuasive communication/ 4078

167 ((tailor* or personali#ed or individuali#ed) adj2 (campaign? or education* or information* or intervention? or initiative? or message? or program* or promotion* or strateg* or study or trial)).tw,kf. 38225

168 motivational interviewing/ or motivational interview*.tw,kf. 6476

169 (psychoeducat* or psycho-educat*).tw,kf. 9413

170 message framing.tw,kf. 553

171 Sex Education/ 9397

172 Prenatal Education/ 362

173 Early Intervention, Educational/ 3585

174 ((women* or mother* or maternal* or prenatal* or pre-natal* or antenatal* or ante-natal* or postnatal* or post-natal* or postpartum* or post-partum*) adj4 (educat* or teach* or train*)).tw,kf. 42946

175 (child health services/ or maternal-child health services/) and pc.fs. 2152

176 (well adj (baby or infant or child) adj care).tw,kf. 824

177 INSERVICE TRAINING/ or staff development/ or communication skill?.tw,kf. 45198

178 Education, Professional/mt [Methods] 841

179 ((educat* or teach or teaching or train*) adj4 (inservice or in-service or personnel* or employe* or staff or worker* or pharmacist* or clinician* or doctor* or physician* or practitioner* or geriatrician* or p?ediatrician* or nurse* or nursing or midwife* or midwive* or health visitor* or health worker? or paramedic* or para-medic* or paraprofessional* or para-professional? or therapist* or counsellor* or counselor* or assistant* or technician* or teacher* or trainer* or leader* or volunteer* or lay or frontline or front line or patient facing)).tw,kf,hw. 286726

180 ((staff or professional) adj development).tw,kf. 16621

181 PUBLIC RELATIONS/ 7190

182 Community-Institutional Relations/ 11003

183 exp Interpersonal Relations/ 354531

184 (community adj4 (mobili#ation or outreach or relation*)).tw,kf. 10676

185 (outreach or out-reach).tw,kf. and educat*.mp. 7422

186 (rais* adj2 awareness adj4 (promotion* or campaign? or intervention* or tool* or strateg*)).tw,kf. 1559

187 Community Networks/ or social support/ or community support/ or psychosocial support systems/ 87495

188 ((communit* or social) adj4 (network* or support)).tw,kf. 109878

189 peer influence/ 821

190 ((advice or advise* or promot* or support* or advocat* or influence* or pressure* or recommend* or led) adj4 (peer* or family or families or friend* or professional* or clinician* or doctor* or physician* or practitioner* or geriatrician* or p?ediatrician* or nurse* or nursing or midwife* or midwive* or health visitor* or health worker* or paramedic* or para-medic* or paraprofessional* or para professional* or therapist* or counsellor* or counselor* or social worker* or leader* or community or communities or teacher* or faith or lay)).tw,kf,hw. 240083

191 Mentors/ 13502

192 (mentor* or role model* or counsel?or?).tw,kf. 42249

193 hotlines/ 2974

194 (champion* or hotline*).tw,kf. 12285

195 Self-Help Groups/ 9674

196 ((group* adj2 support*) or self-help*).tw,kf. 25286

197 *communication/ or (communication.mp. and (mt or pc or px).fs.) 157117

198 DECISION MAKING/ or Decision Making, Shared/ 107245

199 exp Informed Consent/ 44005

200 Choice Behavior/ 35165

201 Consumer Advocacy/ 3481

202 Decision Support Techniques/ 22632

203 (decision* adj2 (aid? or support or tool*)).tw,kf. 43786

204 (toolkit? or tool kit?).tw,kf. 13250

205 (informed adj4 (consent or choice* or decision*)).tw,kf. 69074

206 ((individual* or secondparty or second party or thirdparty or third party or parent* or guardian* or mother* or father* or family or families) adj4 consent*).tw,kf. 7267

207 ((behavio* adj3 chang*) and (campaign? or intervention or program* or strateg* or technique?)).tw,kf. 36276

208 ((tele* or phone*) adj4 (contact* or followup or follow up)).tw,kf. 14642

209 INFORMATION SYSTEMS/ 19540

210 Communications Media/ 1830

211 exp mass media/ 48240

212 Social Marketing/ or public service announcement?.mp. 2965

213 Advertising/ or advertising as topic/ or direct-to-consumer advertising/ 16198

214 advert*.tw,kf. 23473

215 (((print or written or digital) adj (media or material)) or broadside?).tw,kf. 3302

216 *Internet/ 39646

217 Internet-Based Intervention/ 1215

218 Social Media/ or (social media and (campaign or intervention or initiative or message* or promotion* or strateg* or study or trial)).tw,kf. 33412

219 Mobile Applications/ 12380

220 exp Cell Phone/ 23455

221 exp Computers, Handheld/ 13795

222 Medical Informatics Applications/ 2552

223 (radio or television* or tv or broadcast* or podcast* or newspaper* or magazine*).tw,kf. 96752

224 ((health or media) adj4 campaign?).tw,kf. 8819

225 (campaign? adj4 (advert* or banner* or flyer* or handout* or hand-out? or information* or intervention* or leaflet? or letter* or mail* or email or material or messag* or online or pamphlet? or presentation* or program* or promotion* or strateg* or video*)).tw,kf. 8048

226 (promotion* adj4 (advert* or banner* or campaign? or flyer* or handout* or hand-out? or information* or intervention* or leaflet? or letter* or mail* or email or material or messag* or pamphlet? or presentation* or program* or strateg* or video*)).tw,kf. 20912

227 ((universal or population based or national* or nationwide* or statewide* or countrywide* or citywide* or national* or nation wide* or state wide* or country wide* or city wide* or government*) adj4 (campaign or intervention)).tw,kf. 6201

228 (education* adj2 (advert* or banner? or brochure? or campaign? or comm* or flyer* or handout? or hand-out? or information* or intervention* or leaflet? or letter* or mail* or email or material or messag* or online or pamphlet? or poster* or presentation* or program* or promotion* or strateg* or tele* or text* or tool* or video* or www or web or website)).tw,kf. 122937

229 (phone* or telephone* or smartphone* or cellphone* or smartwatch*).ti. 28674

230 ((phone* or tele* or smartphone* or cellphone* or smartwatch) adj3 (based or app* or campaign? or information* or intervention* or messag* or program*)).ab. 40853

231 (mobile* adj3 (based or app* or intervention* or device* or technolog*)).tw,kf. 30541

232 exp video-audio media/ 42504

233 (webinar or webcast or web cast or webconferenc* or web conferenc* or videoconferenc* or video conferenc* or broadcast*).tw,kf. 12719

234 ((app or apps or online or web or website* or internet or digital*) not survey).ti. 157065

235 ((app or apps or online or web or www or website* or internet or digital*) adj3 (based or campaign? or information* or intervention* or messag* or presentation* or program* or tool*)).ab. 113477

236 (twitter or tweet* or blog* or vlog* or pinterest or instagram or facebook or snapchat or tiktok or whatsapp* or chatbot?).tw,kf. 22003

237 (mobile health or mhealth or m-health or ehealth or e-health).ti,kf. 20456

238 ((mobile health or mhealth or m-health or ehealth or e-health) adj3 (based or application* or campaign? or information* or intervention* or messag* or program* or tool*)).ab. 7314

239 Remote Consultation/ 5833

240 remote* consult*.tw,kf. 1248

241 (econsult* or e-consult* or teleconsult* or tele-consult*).tw,kf. 3202

242 (zoom or skype or facetime or face time or digital first or Attend Anywhere or ACCURX or SystmOne).tw,kf. 4888

243 ((complex or factorial or interdisciplinary or inter-disciplinary or multi* component? or multicomponent? or multidisciplin* or multi* disciplin* or multidimension* or multi* dimension* or multifactor* or multi* factor* or multifacet* or multi* facet* or multilevel* or multi* level* or multimodal* or multi* modal* or multiparamet* or multi* paramet* or multiecological or multi* ecological or multistrateg* or multi* strateg*) adj4 (campaign* or intervention? or program* or strateg* or study or system? or trial)).tw,kf. 142359

244 or/144-243 2579899

245 7 and 244 34656

246 ((vaccin* or vaccination? or immuni#ation?) adj4 (educat* or teach* or train*)).tw,kf. 4191

247 ((vaccine? or vaccination? or immuni#ation?) adj4 (communic* or messag* or dialogu* or conversation* or discussion* or negotiation*)).tw,kf. 3476

248 vaccin* information.tw,kf. 1092

249 ((vaccine? or vaccination? or immuni#ation?) adj4 (information* or informed)).tw,kf. and (acceptance or acceptability or attitude? or awareness or (behavi* adj2 chang*) or beliefs or choice? or compliance or consent* or intent* or knowledge or perception? or seeking or trust* or understanding or willingness or campaign or champion* or communication or educat* or encourage* or endorse* or influenc* or nudg* or persua* or promot*).mp. 4462

250 245 or 246 or 247 or 248 or 249 38935

[*Infrastructure Interventions*]

251 "Delivery of Health Care"/og 22540

252 organization.fx. and (immuni#ation? or vaccination?).ti,kf,hw. 4992

253 ((service* or system* or team* or practice* or provider*) adj4 (administ* or organis* or organiz* or coordin* or co ordin* or co-ordin* or logistic* or plan* or structur*)).tw,kf. 243533

254 "Appointments and Schedules"/ 9926

255 appointment*.tw,kf. 34895

256 ((immuni#ation? or vaccination? or revaccination? or reimmuni#ation?) adj4 (scheduling or book* or rebook*)).tw,kf. 169

257 (booking system? or digital registration?).tw,kf. 197

258 "treatment adherence and compliance"/ or patient compliance/ 61915

259 Motivation/ 82009

260 motivat*.ti,kf. or ((motivat* or encourage*) adj4 (vaccination* or immunis* or immuniz*)).ab. 34956

261 ((motivat* or encourage*) adj4 (consumer* or client* or patient* or participant* or individual* or parent* or guardian* or mother* or father* or family or families or adolescent* or teen* or youth* or young* or adult* or old* or elderly or male? or female* or men or women)).tw,kf. 43157

262 Reinforcement, Psychology/ or Reinforcement Schedule/ 24761

263 Reward/ or Token Economy/ 27330

264 Reimbursement, Incentive/ 4820

265 (incentive* or disincentive*).tw,kf. 39437

266 (reward* or token? or voucher?).tw,kf. 76470

267 ((immuni#ation? or vaccination? or revaccination? or reimmuni#ation?) adj4 (reimburs* or pay or payment* or paid)).tw,kf. 416

268 exp Public Assistance/ 88198

269 (social insurance or ((insurance* or social or socio* or tax or welfare) adj4 (allowance* or benefit* or assistance or support or subsidies or claim or claims))).tw,kf. 89730

270 (((cash or financ* or money or monetary or pay or payment* or paid) adj4 (allowance* or benefit* or assistance or support or subsidies or transfer* or claim or claims)) or prepaid or pre-paid or prepayment* or pre-payment*).tw,kf. 24150

271 (punish* or fines or fined or penal* or sanction* or deter or deterred or discourage*).tw,kf. 60360

272 "No Jab No Pay".tw,kf. 25

273 ((document* or proof or prov* or record*) adj4 (immuni#ation? or vaccination?) adj4 (status or up-to-date)).tw,kf. 456

274 ((block* or remov* or take away or withdraw? or with-draw? or withhold* or with-hold* or withheld* or with-held*) adj4 (allowance? or benefit? or ((cash or income or financial or monetary or education* or employment or housing or food) adj (assistance or support)) or social or socio* or subsidies or tax or welfare or (health adj (claim? or insurance?)))).tw,kf. 5008

275 ((block* or remov* or take away or withdraw? or with-draw? or withhold* or with-hold* or withheld* or with-held*) adj4 (childcare or child care or nursery or kindergarten or school?)).tw,kf. 412

276 Mandatory Programs/ 2908

277 ((immuni#ation? or vaccination? or revaccination? or reimmuni#ation?) adj4 (mandat* or compulsory or obligat*)).tw,kf. 2487

278 ((consumer* or client* or patient*) adj4 rights).tw,kf. 5062

279 Choice Behavior/ or Freedom/ 41285

280 or/251-279 880710

281 7 and 280 18540

[*RCT Filter*]

282 exp Randomized Controlled Trial/ 612277

283 Randomized Controlled Trial.pt. 610719

284 Random Allocation/ 107079

285 Controlled Clinical Trial.pt. 95511

286 (randomis* or randomiz*).tw,kf. 857925

287 (RCT or cRCT or "at random" or (random* adj3 (administ* or allocat* or assign* or class* or cluster or crossover or cross-over or control* or determine* or divide* or division or distribut* or expose* or fashion or number* or place* or pragmatic or quasi or recruit* or split or substitut* or treat*))).tw,kf. 765562

288 (intervention or trial).ti,kf. 469365

289 ((intervention? or control* or compar*) adj4 (group? or trial)).ab. 1294769

290 or/282-289 2403570

291 exp Animals/ not Humans/ 5211377

292 290 not 291 2090747

293 limit 292 to yr="2000 -Current" 1736334

294 109 and 293 2936

295 143 and 293 1318

296 250 and 293 3226

297 281 and 293 1262

298 294 or 295 or 296 or 297 5325

[*Countries/topics to remove*]

299 (low income countr* or Afghanistan or Burundi or Burkina Faso or Central African Republic or Eritrea or Ethiopia or Guinea or Gambia or Guinea-Bissau or Liberia or Madagascar or Mali or Mozambique or Malawi or Niger or North Korea or Korean Democratic Republic or Rwanda or Sudan or Sierra Leone or Somalia or South Sudan or Syria or Syrian Arab Republic or Chad or Togo or Uganda or Yemen or Zambia or Algeria or Angola or Bangladesh or Benin or Bhutan or Bolivia or Cote d?Ivoire or Ivory Coast or Cambodia or Cameroon or Congo or Comoros or Cabo Verde or Djibouti or Egypt or Eswatini or El Salvador or Ghana or Haiti or Honduras or India or Indonesia or Iran or Kenya or Kyrgyz Republic or Kiribati or Lao or Lebanon or Lesotho or Mauritania or Micronesia or Mongolia or Morocco or Myanmar or Nepal or Nicaragua or Nigeria or Pakistan or Papua New Guinea or Philippines or Samoa or (Sao Tome and Principe) or Senegal or Solomon Islands or Sri Lanka or Tajikistan or Tanzania or Timor-Leste or Tunisia or Ukraine or Uzbekistan or Vanuatu or Vietnam or (West Bank and Gaza) or Zimbabwe or (Africa and sub-sahara*)).ti. 434074

300 (immunogenic* or immun* genic* or seroepidemiolog* or seroprevalen* or serorespon* or (sero* adj1 (epidemiolog* or prevalen* or respon*))).ti. 36105

301 299 or 300 466821

302 298 not 301 4550

[*Types of Vaccine*]

303 Diphtheria-Tetanus Vaccine/ or Diphtheria-Tetanus-Pertussis Vaccine/ or Diphtheria-Tetanus-Acellular Pertussis Vaccines/ 4593

304 Pertussis Vaccine/ 5523

305 (((diphtheri* or diptheri* or antidiphtheri* or antidiptheri* or whooping cough or pertussis or antipertussis or tetanus or tetani* or antitetanus or antitetani*) adj vaccin*) or ((dpt or dtp or dtwp or di te per or dtap) adj vaccin*)).tw,kf. 7633

306 Measles Vaccine/ or Mumps Vaccine/ or Rubella Vaccine/ or Measles-Mumps-Rubella Vaccine/ 12387

307 ((measles or mumps or rubella or antimeasles or antimumps or antirubella or MMR) adj4 vaccin*).tw,kf. 12450

308 Influenza Vaccines/ 27512

309 (((flu or influenza or antiflu or antiinfluenza) adj4 vaccin*) or (LAIV and vaccin*)).tw,kf. 30114

310 Haemophilus Vaccines/ 3188

311 (((h?emophilus or antih?emophilus) adj4 vaccin*) or (Hib adj vaccin*)).tw,kf. 2511

312 Parainfluenza vaccines/ 57

313 ((parainfluenza or para-influenza) adj4 vaccin*).tw,kf. 216

314 Respiratory Syncytial Virus Vaccines/ 988

315 ((respiratory syncytial virus or rsv) adj4 vaccin*).tw,kf. 1871

316 *Streptococcal Vaccines/ or Pneumococcal Vaccines/ or Heptavalent Pneumococcal Conjugate Vaccine/ 9690

317 (((pneumoni* or pneumococ* or antipneumoni* or antipneumococ*) adj4 vaccin*) or ((PCV* or PPV*) and pneum* and vaccin*) or pneumovax).tw,kf. 13998

318 exp Viral Hepatitis Vaccines/ 13446

319 ((hep* or antihep*) adj2 vaccin*).tw,kf. 11963

320 Meningococcal Vaccines/ 3982

321 ((meningiti* or meningococ* or MenB* or antimeningiti* or antimeningococ* or antiMenB*) adj vaccin*).tw,kf. 2015

322 Poliovirus Vaccines/ or Poliovirus Vaccine, Inactivated/ or Poliovirus Vaccine, Oral/ 7980

323 (((polio* or antipolio*) adj vaccin*) or ((IPV or OPV) and polio* and vaccin*)).tw,kf. 6478

324 Tuberculosis Vaccines/ or BCG Vaccine/ 22665

325 (TB vaccin* or BCG vaccin* or calmette* vaccin* or (tubercul* adj4 vaccin*)).tw,kf. 14134

326 Rotavirus Vaccines/ 2906

327 ((rotavir* or rota-vir* or antirotavir* or antirota-vir*) adj4 vaccin*).tw,kf. 4564

328 Papillomavirus Vaccines/ or Human Papillomavirus Recombinant Vaccine Quadrivalent, Types 6, 11, 16, 18/ 10597

329 (((HPV* or human papillomavir* or antiHPV or antipapillomavir*) adj7 vaccin*) or ((cervical cancer or anticervical cancer) adj vaccin*) or (papillomavir* and cancer and vaccin*) or (Pap* and smear? and vaccin*)).tw,kf. 16412

330 Herpesvirus Vaccines/ or Chickenpox Vaccine/ or Herpes Zoster Vaccine/ 3795

331 (((chickenpox or chicken-pox or varicella or zoster or herpeszoster or shingles) adj4 vaccin*) or ((antichickenpox or antichicken-pox or antivaricella or antizoster or antiherpeszoster or antiherpes-zoster or antishingles) adj4 vaccin*) or ZVL vaccin* or RZV vaccin*).tw,kf. 4539

332 Smallpox Vaccine/ 4107

333 ((smallpox or small pox or variola or antismallpox or antismall-pox or antivariola) adj4 vaccin*).tw,kf. 4452

334 Covid-19 Vaccines/ or chadox1 ncov-19/ or ad26covs1/ or 2019-ncov vaccine mrna-1273/ or bnt162 vaccine/ 25981

335 (((coronavir* or COVID or COVID19 or COVID2019 or 2019-nCoV or 2019nCoV or nCoV-2019 or nCoV2019 or HCoV-19 or HCoV19 or SARS-CoV-2 or SARSCo-V2 or SARS-CoV2 or SARSCoV2 or SARSCoV-2 or SARS2 or severe acute respiratory syndrome) adj4 vaccin*) or mRNA vaccines).tw,kf. 45027

336 (Monkeypox/ or Monkeypox virus/) and Vaccines/ 19

337 ((monkey pox or monkeypox or antimonkey pox or antimonkeypox) adj4 vaccin*).tw,kf. 412

338 or/303-337 211463

339 (108 and 338 and 293) not 298 234

340 (139 and 338 and 293) not 298 106

341 (244 and 338 and 293) not 298 218

342 (280 and 338 and 293) not 298 71

343 339 or 340 or 341 or 342 497

344 343 not 301 433

345 302 or 344 4983

[*Date limited:* Sept-2023 to 8-Apr-2024 (initial searches from 2000 onwards)]

346 (202309* or 202310* or 202311* or 202312* or 2024*).ep. 787571

347 (2023 09* or 2023 10* or 2023 11* or 2023 12* or 2024*).dp. 711986

348 ("2023 Sep*" or "2023 Oct*" or "2023 Nov*" or "2023 Dec*" or "2024 Jan*" or "2024 Feb*" or "2024 Mar*" or "2024 Apr*").dp. 814982

349 ("2023/09*" or "2023/10*" or "2023/11*" or "2023/12*" or "2024*").ez,dt. 466889

350 346 or 347 or 348 or 349 1119567

351 345 and 350 412

352 2024*.yr. 575851

353 346 or 347 or 348 or 349 or 352 1119567

354 345 and 353 412

355 (2022* or 2023* or 2024*).yr,dp,dt,ep,ez. 3882600

356 345 and 355 1451

Key to date fields:

dp: date of publication

dt: create date

ep: electronic date of publication

ez: Entrez date

yr: year of publication

1. **Search strategy for economic data**

Ovid MEDLINE(R) ALL - Search

| Health Economics Filter | |
| --- | --- |
| 1 | *Economics/ |
| 2 | Value of life/ |
| 3 | exp "costs and cost analysis"/ |
| 4 | exp economics, hospital/ |
| 5 | exp economics, medical/ |
| 6 | exp "fees and charges"/ |
| 7 | exp budgets/ |
| 8 | budget*.tw,kf. |
| 9 | intervention costs.tw,kf. |
| 10 | (cost? per adj2 (infant or child or adolescent or adult or man or woman or male or female)).tw,kf. |
| 11 | economic*.ti. |
| 12 | (cost* adj2 (effective* or utilit* or benefit* or minimi* or unit* or estimat* or variable*)).ab. |
| 13 | (value adj2 (money or monetary)).tw,kf. |
| 14 | or/1-13 |
| 15 | exp Health Care Costs/ |
| 16 | exp Drug Costs/ |
| 17 | exp "Cost of Illness"/ |
| 18 | Health Expenditures/ |
| 19 | exp Drug Utilization/ |
| 20 | exp Cost-Benefit Analysis/ |
| 21 | (cost? adj2 (illness or disease or sickness or health care or healthcare or treatment or direct or indirect or medical or resource)).tw,kf. |
| 22 | (burden? adj2 economic*).tw,kf. |
| 23 | (utili?ation adj2 (health or medical or resource)).tw,kf. |
| 24 | (out-of-pocket adj2 (payment? or expenditure? or cost? or spending or expense?)).tw,kf. |
| 25 | (expenditure? adj3 (health or direct or indirect)).tw,kf. |
| 26 | (healthcare cost* or health care cost* or healthcare utili?ation or health care utili?ation or cost of illness).tw,kf. |
| 27 | (value adj2 (money or monetary)).tw,kf. |
| 28 | (cost* adj2 (effective* or utilit* or benefit* or analy* or outcome or outcomes)).tw,kf. |
| 29 | or/15-28 |
| 30 | quality-adjusted life years/ |
| 31 | ((quality or index) adj2 (wellbeing or well-being)).tw,kf. |
| 32 | disability adjusted life.tw,kf. |
| 33 | (qaly* or daly*).tw,kf. |
| 34 | (quality adjusted or adjusted life year* or quality adjusted life year*).tw,kf. |
| 35 | (eq-5d or eq5d or eq-5 or eq5 or euroqual or euro qual or euro qual5d or euroqual5d or euro qol or euroqol or euro qol5d or euroqol5d or euro quol or euroquol or euro quol5d or euroquol5d or eur qol or eurqol or eur qol5d or eur qol5d or eur?qul or eur?qul5d or euro* quality of life or european qol).ti,ab,kf. |
| 36 | (euro* adj3 (5 d or 5d or 5 dimension* or 5dimension* or 5 domain* or 5domain*)).tw,kf. |
| 37 | (qol or hql* or hqol* or h qol* or hrqol* or hr qol* or (quality adj2 life)).ti,kf. and ((qol or hql* or hqol* or h qol* or hrqol* or hr qol* or quality of life) adj2 (increase* or decrease* or improv* or declin* or reduc* or high* or low* or effect or effects or worse or score or scores or change? or impact? or impacted or deteriorate*)).ab. |
| 38 | *"Quality of Life"/ and ((qol or hql* or hqol* or h qol* or hrqol* or hr qol* or quality of life) adj2 (increase* or decrease* or improv* or declin* or reduc* or high* or low* or effect or effects or worse or score or scores or change? or impact? or impacted or deteriorate*)).ab. |
| 39 | "quality of life"/ and ((quality of life or qol) adj (score? or measure?)).tw,kf. |
| 40 | "quality of life"/ and economics.fs. |
| 41 | "quality of life"/ and (health adj3 status).tw,kf. |
| 42 | ((quality adj2 (life? or lives)) or qol).tw,kf. and Cost-Benefit Analysis/ |
| 43 | "quality of life"/ and ((quality and (life? or lives)) or qol).ti. |
| 44 | "quality of life"/ and ((quality or qol) adj3 (improv* or chang*)).tw,kf. |
| 45 | "quality of life"/ and health related quality.tw,kf. |
| 46 | Cost-Benefit Analysis/ and (cost-effectiveness ratio* and (perspective* or life expectanc*)).tw,kf. |
| 47 | (health utility* or utility score* or disutilit*).tw,kf. |
| 48 | (utilities or (utilit$ adj3 (score? or value* or health* or cost* or analys* or measur* or disease* or mean or gain or gains or index or indices))).tw,kf. |
| 49 | (hui or hui1 or hui-1 or hui2 or hui-2 or hui3 or hui-3).tw,kf. |
| 50 | health* year* equivalent*.tw,kf. |
| 51 | (willingness to pay or time tradeoff or time trade off or tto or standard gamble*).tw,kf. |
| 52 | (sf36* or sf-36* or sf 36 or sf6 or sf 6 or sf-6 or sf6d or sf 6d or sf-6d or sf8 or sf-8 or sf 8 or sf12 or sf-12 or sf 12 or sf16 or sf-16 or sf 16 or sf20 or sf-20 or sf 20 or sf thirtysix or sf thirty six).tw,kf. |
| 53 | (visual analog* scale* or EQ-VAS).tw,kf. |
| 54 | or/30-53 |
| 55 | exp models, economic/ |
| 56 | (markov* or monte carlo).tw,kf. |
| 57 | econom* model*.tw,kf. |
| 58 | ((value adj2 information analysis) or (expected value adj3 perfect information) or (expected value adj3 sampl* information)).tw,kf. |
| 59 | (microsimulation? or micro-simulation?).tw,kf. |
| 60 | discrete event? simulation?.tw,kf. |
| 61 | discrete choice experiment*.tw,kf. |
| 62 | or/55-61 |
| 63 | immunization/ec or immunization schedule/ec or vaccination/ec or mass vaccination/ec |
| 64 | 14 or 29 or 54 or 62 or 63 |

| Interventions by type: Access; Reminders; Education; Infrastructure | |
| --- | --- |
| 65 | (*Vaccination/ or *Immunization/) and "Organization & Administration".fs. |
| 66 | *Preventive Health Services/ |
| 67 | Health Services Accessibility/ |
| 68 | ("after hours" or "out of hours" or (extend* adj2 hours)).tw,kf. |
| 69 | ((extend* or weekend? or early or earlier or late or later or evening? or commuter?) adj4 (clinic? or service? or appointment? or session?)).tw,kf. |
| 70 | Mobile Health Units/ |
| 71 | ((mobile adj4 (clinic? or health unit? or health care or healthcare or health van? or hospital? or site or sites)) or field hospital?).tw,kf. |
| 72 | (temporary adj4 (centre? or center? or clinic? or facility or facilities or health care or healthcare or health unit? or service? or site or sites)).tw,kf. |
| 73 | (walkin or "walk in" or popup or pop-up or popin or "pop in" or "pop by" or "drop by").tw,kf. |
| 74 | ((dropin or "drop in") adj4 (centre? or center? or clinic? or facility or facilities or health unit? or service? or site or sites)).tw,kf. |
| 75 | (drive-in or drive-through).tw,kf. |
| 76 | (supermarket? or grocery store? or drug store? or instore? or in-store? or shopping cent* or retail unit? or mall or malls or out-of-town).tw,kf. |
| 77 | (sporting venue? or stadium? or stadia or arenas).tw,kf. |
| 78 | ((urban or rural) adj4 (access* or availability)).tw,kf. |
| 79 | (escort* or group visit*).tw,kf. |
| 80 | (health* adj (visit* or supervis*)).tw,kf. |
| 81 | Safety-net Providers/ |
| 82 | ((safety-net or safetynet) adj3 (clinic? or hospital? or provider? or system? or health care or healthcare)).tw,kf. |
| 83 | exp Community Participation/ |
| 84 | Community Health Centers/ or Community Health Services/ or exp Community Health Nursing/ or Community Mental Health Services/ or Community Networks/ |
| 85 | Public Health Nursing/ |
| 86 | ((community or public or civic or communal or municipal) adj4 (facility or facilities or provider? or setting? or venue* or located or location? or building* or regional* or clinic? or hall or halls or centre* or center* or space or spaces or site or sites)).tw,kf. |
| 87 | (communit* adj4 (advoc* or awareness or champion* or compliance or educat* or engag* or involv* or participat* or uptake?)).tw,kf. |
| 88 | (community group? or (communit* adj4 (activ* or campaign* or implement* or intervention or policy or policies or program* or pilot or strategy or strategies or workshop?))).tw,kf. |
| 89 | ((community or community health*) adj (care or unit? or nurs* or service? or volunteer*)).tw,kf. |
| 90 | (community adj4 (dwelling or based or delivered or located or location? or led or run or set or setting?)).tw,kf. |
| 91 | (communit* adj4 (rural or urban or inner city)).tw,kf. |
| 92 | Substance Abuse Treatment Centers/ |
| 93 | ((drug? or substance?) adj4 (abuse or treat* or rehab*) adj4 (centre? or center? or clinic? or facility or facilities or health care or healthcare or health unit? or service? or site or sites)).tw,kf. |
| 94 | ((family or families or carer? or parent* or office or work*) adj (based or delivered)).tw,kf. |
| 95 | Community Pharmacy Services/ or Pharmacy/ |
| 96 | (pharmacy or pharmacies or pharmacist?).tw,kf. |
| 97 | *General Practice/ or *Family Practice/ |
| 98 | (((general practi* or family practi* or primary care) adj7 (intervention? or program* or project? or service? or study or trial)) and (access* or uptake? or cover* or rate or rates or visit*)).tw,kf. |
| 99 | Family Nursing/ or Maternal-Child Nursing/ |
| 100 | Prenatal Care/ or Perinatal care/ or Maternal Child Health centers/ |
| 101 | Peripartum Period/ or Postpartum Period/ |
| 102 | ((prenatal or pre-natal or antenatal or ante-natal or pregnan* or perinatal or peri-natal or postpartum or post-partum) adj4 (care or centre? or center? or clinic? or facility or facilities or health care or healthcare or health unit? or service? or site or sites)).tw,kf. |
| 103 | (mother? adj4 (baby or babies) adj4 (care or centre? or center? or clinic? or facility or facilities or health care or healthcare or health unit? or service? or site or sites)).tw,kf. |
| 104 | ((midwif* or widwiv*) adj4 (based or delivered or led or run or care or centre? or center? or clinic? or facility or facilities or health care or healthcare or health unit? or service? or site or sites)).tw,kf. |
| 105 | (mother? adj4 program*).tw,kf. |
| 106 | (well-child adj (care or clinic)).tw,kf. |
| 107 | ((women adj2 Infants adj2 children) or (WIC adj3 (program* or service? or support*))).tw,kf. |
| 108 | exp Child Day Care Centers/ |
| 109 | ((child or children* or day or daycare) adj4 (centre? or center?)).tw,kf. |
| 110 | Schools, Nursery/ |
| 111 | ((nursery or nurseries or kindergarten* or preschool* or pre-school*) adj4 (based or delivered or located or location? or led or run or set or setting?)).tw,kf. |
| 112 | ((nursery or nurseries or kindergarten* or preschool* or pre-school*) adj4 (clinic or clinics or health* or nurs* or program* or vaccin* or immuni#ation?)).tw,kf. |
| 113 | ((nursery or nurseries or kindergarten* or preschool* or pre-school*) adj7 (accessibility or "access to" or uptake? or cover* or rate or rates)).tw,kf. |
| 114 | (play school* or play group?).tw,kf. |
| 115 | School Health Services/ |
| 116 | Schools/ and (exp vaccines/ or Vaccination/) |
| 117 | ((school? or highschool?) adj4 (based or delivered or located or location? or led or run or set or setting?)).tw,kf. |
| 118 | (school? adj4 (clinic? or health care or healthcare or health unit? or nurs* or vaccin* or immuni#ation?)).tw,kf. |
| 119 | (school? adj7 (accessibility or "access to" or uptake? or cover* or rate or rates)).tw,kf. |
| 120 | ((universit* or colleg*) adj4 (based or delivered or located or location? or led or run or set or setting? or students)).tw,kf. |
| 121 | ((universit* or colleg*) adj4 (clinic? or health care or healthcare or health unit? or nurs* or program* or vaccin* or immuni#ation?)).tw,kf. |
| 122 | (library adj4 (based or delivered or located or location? or led or run or set or setting?)).tw,kf. |
| 123 | (public library or (library adj4 (clinic? or health care or healthcare or health unit? or nurs* or program* or based or delivered or setting? or vaccin* or immuni#ation?))).tw,kf. |
| 124 | ((leisure or recreation or sports) adj4 (centre? or center? or facility or facilities)).tw,kf. |
| 125 | (sports hall? or gym or gyms or gymnasium?).tw,kf. |
| 126 | (faith adj2 organi#ation?).tw,kf. |
| 127 | ((religion or religious) adj4 (building? or centre? or center? or facility or facilities or delivered or located or location? or led or run or set or setting?)).tw,kf. |
| 128 | ((faith or church* or mosque? or synagogue?) adj4 (clinic? or health care or healthcare or nurs* or program* or based or delivered or setting? or vaccin* or immunis* or immuniz*)).tw,kf. |
| 129 | Transcultural Nursing/ |
| 130 | "Religion and Medicine"/ |
| 131 | home care services/ or home health nursing/ or home nursing/ |
| 132 | House Calls/ |
| 133 | (housecall? or house call? or outreach or out-reach or (door adj2 door) or ((home or homes) adj4 visit*) or (home adj4 (immuniz* or immunis* or vaccinat*))).tw,kf. |
| 134 | adult day care centers/ or Senior Centers/ |
| 135 | orphanages/ or Foster Home Care/ |
| 136 | (orphange? or foster care or foster home* or kinship care or children* home*).tw,kf. |
| 137 | exp Education, Special/ |
| 138 | (special adj4 (education or school*)).tw,kf. |
| 139 | halfway houses/ |
| 140 | exp Homeless Persons/ |
| 141 | (homeless* or home less* or hostel? or ((halfway or half-way) adj (house? or home?))).tw,kf. |
| 142 | Ambulatory Care Facilities/ |
| 143 | Emergency Service, Hospital/ |
| 144 | Outpatient Clinics, Hospital/ |
| 145 | ((outpatient? or out-patient? or out-of-hospital) adj7 (intervention? or program* or project? or service? or study or trial)).tw,kf. |
| 146 | ((outpatient? or out-patient?) adj4 (centre? or center? or clinic? or unit? or visit*)).tw,kf. |
| 147 | (ambulatory care or ambulance? or paramed* or para-med* or paraprofessional? or para-professional? or emergency department? or (accident* adj2 emergenc*) or ED visit*).tw,kf. |
| 148 | (ED immunis* or ED immuniz* or ED vaccinat*).tw,kf. |
| 149 | (case management or case manager? or case worker?).mp. |
| 150 | (interpreter* or translator*).tw,kf. |
| 151 | (social adj (support or work*)).mp. |
| 152 | Vaccination/nu or vaccin* manager?.tw,kf. |
| 153 | Occupational Health/ |
| 154 | ((work adj2 (based or delivered or place? or site or sites)) or workbased or workplace? or worksite?).tw,kf. |
| 155 | ((vaccinat* or immuni#ation? or reimmuni#ation?) adj4 (occupational health or employee? or employer? or frontline or front-line or personnel or provider? or staff or worker? or work force or workforce or "at work")).tw,kf. |
| 156 | (opportun* adj (vaccination? or immuni#ation?)).tw,kf. |
| 157 | (opportun* adj4 (catch-up or catchup or boost* or revaccinat* or reimmunis* or reimmuniz* or re-vaccinati* or re-immunis* or re-immuniz*)).tw,kf. |
| 158 | or/65-157 |
| 159 | Reminder Systems/ |
| 160 | ((remind* or recall* or messag* or alert*) adj4 system?).tw,kf. |
| 161 | (reminder? or prompts or prompting or prompted or nudge?).tw,kf. |
| 162 | ((immuni* or vaccin*) adj4 (recall* or remind*)).tw,kf. |
| 163 | (recall* adj4 (alert* or appointment? or based or boost* or calendar or campaign* or central* or correspond* or initiative? or intervention? or invit* or letter? or mail or email or message? or notice? or phone? or postal or "by post" or program* or prompt* or regist* or remind*)).tw,kf. |
| 164 | (remind* adj4 (alert* or appointment? or based or boost* or calendar or campaign* or central* or correspond* or initiative? or intervention? or invit* or letter? or mail or email or message? or notice? or phone? or postal or "by post" or program* or prompt* or regist* or recall*)).tw,kf. |
| 165 | ((recall* or remind*) adj4 (app* or auto* or computer* or digital* or electronic or tele* or SMS or text*)).tw,kf. |
| 166 | ((recall* or remind*) adj4 (adults or patients or individuals or parent* or carer? or mothers or fathers or family or families or men or women)).tw,kf. |
| 167 | ((recall* or remind*) adj4 (employee? or employer? or frontline or front-line or personnel or provider? or staff or worker? or work force or workforce)).tw,kf. |
| 168 | (remind* or recall*).ti. |
| 169 | ((auto* or computer*) adj4 (alert* or messag*)).tw,kf. |
| 170 | (autodial* or (auto* adj4 (call* or dial* or tele*))).tw,kf. |
| 171 | correspondence as topic/ |
| 172 | postal service/ |
| 173 | invitation?.tw,kf. |
| 174 | ((immuni* or vaccin*) adj4 letter?).tw,kf. |
| 175 | (communication? and (vaccin* or Immuni#ation*)).ti,kf,hw. |
| 176 | (mail* out? or circulars).tw,kf. |
| 177 | electronic mail/ or text messaging/ |
| 178 | posters as topic/ or poster?.tw,kf. |
| 179 | pamphlets/ or (pamphlet? or leaflet? or brochure?).tw,kf. |
| 180 | (recei* adj4 (vaccin* or immuni#ation?) adj4 (alert* or information or messag*)).tw,kf. |
| 181 | decision support system?.mp. |
| 182 | (decision adj4 making adj4 (auto* or system? or technique?)).tw,kf. |
| 183 | ((activat* or auto* or check* or review or update or up-to-date) adj3 (health* or medical or patient?) adj3 record?).tw,kf. |
| 184 | (immunosurveillance or immuno-surveillance).mp. |
| 185 | (postcard* or post* card*).tw,kf. |
| 186 | (vaccin* adj4 (text? or tele* or recall* or remind* or prompt* or postcard* or post* card* or postal or nudg* or messag* or mail or email? or letter? or invit* or autodial* or auto* dial* or alert*)).tw,kf. |
| 187 | Reminder Systems/ and (Primary Prevention/ or Preventive Health Services/) |
| 188 | or/159-187 |
| 189 | HEALTH PROMOTION/ or (public health adj (campaign? or message?)).mp. |
| 190 | Health Education/ |
| 191 | (health adj (education or promotion)).tw,kf. |
| 192 | Knowledge/ or Patient Medication Knowledge/ |
| 193 | Health Knowledge, Attitudes, Practice/ and (uptake? or coverage).mp. |
| 194 | Patient Acceptance of Health Care/ and (uptake? or coverage).mp. |
| 195 | Education/ |
| 196 | Health Literacy/ |
| 197 | Patient Education as Topic/ |
| 198 | consumer health information/ |
| 199 | National Health Programs/ |
| 200 | Government Publications as Topic/ |
| 201 | Health Fairs/ or health fair?.tw,kf. |
| 202 | (education* adj4 (campaign? or information* or intervention? or initiative? or message? or program* or promotion* or strateg* or tailor* or tool* or target* or study or trial)).tw,kf. |
| 203 | curriculum/ or curricul*.tw,kf. |
| 204 | (literacy or illiterat*).tw,kf. |
| 205 | (campaign or education*).ti. |
| 206 | ((client? or consumer? or patient? or public) adj4 (educat* or teach* or train*)).tw,kf. |
| 207 | (educat* and inform*).ti,kf,hw. or "educat* and inform*".ab. |
| 208 | (fact sheet? or factual information or informational).tw,kf. |
| 209 | Information Seeking Behavior/ |
| 210 | Information Dissemination/ |
| 211 | persuasive communication/ |
| 212 | ((tailor* or personali#ed or individuali#ed) adj2 (campaign? or education* or information* or intervention? or initiative? or message? or program* or promotion* or strateg* or study or trial)).tw,kf. |
| 213 | motivational interviewing/ or motivational interview*.tw,kf. |
| 214 | (psychoeducat* or psycho-educat*).tw,kf. |
| 215 | message framing.tw,kf. |
| 216 | Sex Education/ |
| 217 | Prenatal Education/ |
| 218 | Early Intervention, Educational/ |
| 219 | ((women* or mother* or maternal* or prenatal* or pre-natal* or antenatal* or ante-natal* or postnatal* or post-natal* or postpartum* or post-partum*) adj4 (educat* or teach* or train*)).tw,kf. |
| 220 | (child health services/ or maternal-child health services/) and pc.fs. |
| 221 | (well adj (baby or infant or child) adj care).tw,kf. |
| 222 | INSERVICE TRAINING/ or staff development/ or communication skill?.tw,kf. |
| 223 | Education, Professional/mt [Methods] |
| 224 | ((educat* or teach or teaching or train*) adj4 (inservice or in-service or personnel* or employe* or staff or worker* or pharmacist* or clinician* or doctor* or physician* or practitioner* or geriatrician* or p?ediatrician* or nurse* or nursing or midwife* or midwive* or health visitor* or health worker? or paramedic* or para-medic* or paraprofessional* or para-professional? or therapist* or counsellor* or counselor* or assistant* or technician* or teacher* or trainer* or leader* or volunteer* or lay or frontline or front line or patient facing)).tw,kf,hw. |
| 225 | ((staff or professional) adj development).tw,kf. |
| 226 | PUBLIC RELATIONS/ |
| 227 | Community-Institutional Relations/ |
| 228 | exp Interpersonal Relations/ |
| 229 | (community adj4 (mobili#ation or outreach or relation*)).tw,kf. |
| 230 | (outreach or out-reach).tw,kf. and educat*.mp. |
| 231 | (rais* adj2 awareness adj4 (promotion* or campaign? or intervention* or tool* or strateg*)).tw,kf. |
| 232 | Community Networks/ or social support/ or community support/ or psychosocial support systems/ |
| 233 | ((communit* or social) adj4 (network* or support)).tw,kf. |
| 234 | peer influence/ |
| 235 | ((advice or advise* or promot* or support* or advocat* or influence* or pressure* or recommend* or led) adj4 (peer* or family or families or friend* or professional* or clinician* or doctor* or physician* or practitioner* or geriatrician* or p?ediatrician* or nurse* or nursing or midwife* or midwive* or health visitor* or health worker* or paramedic* or para-medic* or paraprofessional* or para professional* or therapist* or counsellor* or counselor* or social worker* or leader* or community or communities or teacher* or faith or lay)).tw,kf,hw. |
| 236 | Mentors/ |
| 237 | (mentor* or role model* or counsel?or?).tw,kf. |
| 238 | hotlines/ |
| 239 | (champion* or hotline*).tw,kf. |
| 240 | Self-Help Groups/ |
| 241 | ((group* adj2 support*) or self-help*).tw,kf. |
| 242 | *communication/ or (communication.mp. and (mt or pc or px).fs.) |
| 243 | *DECISION MAKING/ or Decision Making, Shared/ |
| 244 | exp Informed Consent/ |
| 245 | Choice Behavior/ |
| 246 | Consumer Advocacy/ |
| 247 | *Decision Support Techniques/ |
| 248 | (decision* adj2 (aid? or support or tool*)).tw,kf. |
| 249 | (toolkit? or tool kit?).tw,kf. |
| 250 | (informed adj4 (consent or choice* or decision*)).tw,kf. |
| 251 | ((individual* or secondparty or second party or thirdparty or third party or parent* or guardian* or mother* or father* or family or families) adj4 consent*).tw,kf. |
| 252 | ((behavio* adj3 chang*) and (campaign? or intervention or program* or strateg* or technique?)).tw,kf. |
| 253 | ((tele* or phone*) adj4 (contact* or followup or follow up)).tw,kf. |
| 254 | INFORMATION SYSTEMS/ |
| 255 | Communications Media/ |
| 256 | exp mass media/ |
| 257 | Social Marketing/ or public service announcement?.mp. |
| 258 | Advertising/ or advertising as topic/ or direct-to-consumer advertising/ |
| 259 | advert*.tw,kf. |
| 260 | (((print or written or digital) adj (media or material)) or broadside?).tw,kf. |
| 261 | *Internet/ |
| 262 | Internet-Based Intervention/ |
| 263 | Social Media/ or (social media and (campaign or intervention or initiative or message* or promotion* or strateg* or study or trial)).tw,kf. |
| 264 | Mobile Applications/ |
| 265 | exp Cell Phone/ |
| 266 | exp Computers, Handheld/ |
| 267 | Medical Informatics Applications/ |
| 268 | (radio or television* or tv or broadcast* or podcast* or newspaper* or magazine*).tw,kf. |
| 269 | ((health or media) adj4 campaign?).tw,kf. |
| 270 | (campaign? adj4 (advert* or banner* or flyer* or handout* or hand-out? or information* or intervention* or leaflet? or letter* or mail* or email or material or messag* or online or pamphlet? or presentation* or program* or promotion* or strateg* or video*)).tw,kf. |
| 271 | (promotion* adj4 (advert* or banner* or campaign? or flyer* or handout* or hand-out? or information* or intervention* or leaflet? or letter* or mail* or email or material or messag* or pamphlet? or presentation* or program* or strateg* or video*)).tw,kf. |
| 272 | ((universal or population based or national* or nationwide* or statewide* or countrywide* or citywide* or national* or nation wide* or state wide* or country wide* or city wide* or government*) adj4 (campaign or intervention)).tw,kf. |
| 273 | (education* adj2 (advert* or banner? or brochure? or campaign? or comm* or flyer* or handout? or hand-out? or information* or intervention* or leaflet? or letter* or mail* or email or material or messag* or online or pamphlet? or poster* or presentation* or program* or promotion* or strateg* or tele* or text* or tool* or video* or www or web or website)).tw,kf. |
| 274 | (phone* or telephone* or smartphone* or cellphone* or smartwatch*).ti. |
| 275 | ((phone* or tele* or smartphone* or cellphone* or smartwatch) adj3 (based or app* or campaign? or information* or intervention* or messag* or program*)).ab. |
| 276 | (mobile* adj3 (based or app* or intervention* or device* or technolog*)).tw,kf. |
| 277 | exp video-audio media/ |
| 278 | (webinar or webcast or web cast or webconferenc* or web conferenc* or videoconferenc* or video conferenc* or broadcast*).tw,kf. |
| 279 | ((app or apps or online or web or website* or internet or digital*) not survey).ti. |
| 280 | ((app or apps or online or web or www or website* or internet or digital*) adj3 (based or campaign? or information* or intervention* or messag* or presentation* or program* or tool*)).ab. |
| 281 | (twitter or tweet* or blog* or vlog* or pinterest or instagram or facebook or snapchat or tiktok or whatsapp* or chatbot?).tw,kf. |
| 282 | (mobile health or mhealth or m-health or ehealth or e-health).ti,kf. |
| 283 | ((mobile health or mhealth or m-health or ehealth or e-health) adj3 (based or application* or campaign? or information* or intervention* or messag* or program* or tool*)).ab. |
| 284 | Remote Consultation/ |
| 285 | remote* consult*.tw,kf. |
| 286 | (econsult* or e-consult* or teleconsult* or tele-consult*).tw,kf. |
| 287 | (zoom or skype or facetime or face time or digital first or Attend Anywhere or ACCURX or SystmOne).tw,kf. |
| 288 | ((complex or factorial or interdisciplinary or inter-disciplinary or multi* component? or multicomponent? or multidisciplin* or multi* disciplin* or multidimension* or multi* dimension* or multifactor* or multi* factor* or multifacet* or multi* facet* or multilevel* or multi* level* or multimodal* or multi* modal* or multiparamet* or multi* paramet* or multiecological or multi* ecological or multistrateg* or multi* strateg*) adj4 (campaign* or intervention? or program* or strateg* or study or system? or trial)).tw,kf. |
| 289 | ((vaccin* or vaccination? or immuni#ation?) adj4 (educat* or teach* or train*)).tw,kf. |
| 290 | ((vaccine? or vaccination? or immuni#ation?) adj4 (communic* or messag* or dialogu* or conversation* or discussion* or negotiation*)).tw,kf. |
| 291 | vaccin* information.tw,kf. |
| 292 | ((vaccine? or vaccination? or immuni#ation?) adj4 (information* or informed)).tw,kf. and (acceptance or acceptability or attitude? or awareness or (behavi* adj2 chang*) or beliefs or choice? or compliance or consent* or intent* or knowledge or perception? or seeking or trust* or understanding or willingness or campaign or champion* or communication or educat* or encourage* or endorse* or influenc* or nudg* or persua* or promot*).mp. |
| 293 | or/189-292 |
| 294 | "Delivery of Health Care"/og |
| 295 | ((service* or system* or team* or practice* or provider*) adj4 (administ* or organis* or organiz* or coordin* or co ordin* or co-ordin* or logistic* or plan* or structur*)).tw,kf. |
| 296 | "Appointments and Schedules"/ |
| 297 | appointment*.tw,kf. |
| 298 | ((immuni#ation? or vaccination? or revaccination? or reimmuni#ation?) adj4 (scheduling or book* or rebook*)).tw,kf. |
| 299 | (booking system? or digital registration?).tw,kf. |
| 300 | "treatment adherence and compliance"/ or patient compliance/ |
| 301 | Motivation/ |
| 302 | motivat*.ti,kf. or ((motivat* or encourage*) adj4 (vaccination* or immunis* or immuniz*)).ab. |
| 303 | ((motivat* or encourage*) adj4 (consumer* or client* or patient* or participant* or individual* or parent* or guardian* or mother* or father* or family or families or adolescent* or teen* or youth* or young* or adult* or old* or elderly or male? or female* or men or women)).tw,kf. |
| 304 | Reinforcement, Psychology/ or Reinforcement Schedule/ |
| 305 | Reward/ or Token Economy/ |
| 306 | Reimbursement, Incentive/ |
| 307 | (incentive* or disincentive*).tw,kf. |
| 308 | (reward* or token? or voucher?).tw,kf. |
| 309 | ((immuni#ation? or vaccination? or revaccination? or reimmuni#ation?) adj4 (reimburs* or pay or payment* or paid)).tw,kf. |
| 310 | exp Public Assistance/ |
| 311 | (social insurance or ((insurance* or social or socio* or tax or welfare) adj4 (allowance* or benefit* or assistance or support or subsidies or claim or claims))).tw,kf. |
| 312 | (((cash or financ* or money or monetary or pay or payment* or paid) adj4 (allowance* or benefit* or assistance or support or subsidies or transfer* or claim or claims)) or prepaid or pre-paid or prepayment* or pre-payment*).tw,kf. |
| 313 | (punish* or fines or fined or penal* or sanction* or deter or deterred or discourage*).tw,kf. |
| 314 | "No Jab No Pay".tw,kf. |
| 315 | ((document* or proof or prov* or record*) adj4 (immuni#ation? or vaccination?) adj4 (status or up-to-date)).tw,kf. |
| 316 | ((block* or remov* or take away or withdraw? or with-draw? or withhold* or with-hold* or withheld* or with-held*) adj4 (allowance? or benefit? or ((cash or income or financial or monetary or education* or employment or housing or food) adj (assistance or support)) or social or socio* or subsidies or tax or welfare or (health adj (claim? or insurance?)))).tw,kf. |
| 317 | ((block* or remov* or take away or withdraw? or with-draw? or withhold* or with-hold* or withheld* or with-held*) adj4 (childcare or child care or nursery or kindergarten or school?)).tw,kf. |
| 318 | Mandatory Programs/ |
| 319 | ((immuni#ation? or vaccination? or revaccination? or reimmuni#ation?) adj4 (mandat* or compulsory or obligat*)).tw,kf. |
| 320 | ((consumer* or client* or patient*) adj4 rights).tw,kf. |
| 321 | Choice Behavior/ or Freedom/ |
| 322 | or/294-321 |
| 323 | 158 or 188 or 293 or 322 |
| 324 | 64 and 323 |

| Types of Vaccine | |
| --- | --- |
| 325 | Diphtheria-Tetanus Vaccine/ or Diphtheria-Tetanus-Pertussis Vaccine/ or Diphtheria-Tetanus-Acellular Pertussis Vaccines/ |
| 326 | Pertussis Vaccine/ |
| 327 | (((diphtheri* or diptheri* or antidiphtheri* or antidiptheri* or whooping cough or pertussis or antipertussis or tetanus or tetani* or antitetanus or antitetani*) adj vaccin*) or ((dpt or dtp or dtwp or di te per or dtap) adj vaccin*)).ti,kf. |
| 328 | Measles Vaccine/ or Mumps Vaccine/ or Rubella Vaccine/ or Measles-Mumps-Rubella Vaccine/ |
| 329 | ((measles or mumps or rubella or antimeasles or antimumps or antirubella or MMR) adj4 vaccin*).ti,kf. |
| 330 | *Influenza Vaccines/ or (Influenza Vaccines/ and ec.fs.) |
| 331 | (((flu or influenza or antiflu or antiinfluenza) adj4 vaccin*) or (LAIV and vaccin*)).ti,kf. |
| 332 | *Haemophilus Vaccines/ or (Haemophilus Vaccines/ and ec.fs.) |
| 333 | (((h?emophilus or antih?emophilus) adj4 vaccin*) or (Hib adj vaccin*)).ti,kf. |
| 334 | *Parainfluenza vaccines/ or (Parainfluenza vaccines/ and ec.fs.) |
| 335 | ((parainfluenza or para-influenza) adj4 vaccin*).ti,kf. |
| 336 | Respiratory Syncytial Virus Vaccines/ |
| 337 | ((respiratory syncytial virus or rsv) adj4 vaccin*).ti,kf. |
| 338 | *Streptococcal Vaccines/ or *Pneumococcal Vaccines/ or *Heptavalent Pneumococcal Conjugate Vaccine/ or ((Streptococcal Vaccines/ or Pneumococcal Vaccines/ or Heptavalent Pneumococcal Conjugate Vaccine/) and ec.fs.) |
| 339 | (((pneumoni* or pneumococ* or antipneumoni* or antipneumococ*) adj4 vaccin*) or ((PCV* or PPV*) and pneum* and vaccin*) or pneumovax).ti,kf. |
| 340 | exp *Viral Hepatitis Vaccines/ or (exp Viral Hepatitis Vaccines/ and ec.fs.) |
| 341 | ((hep* or antihep*) adj2 vaccin*).ti,kf. |
| 342 | Meningococcal Vaccines/ |
| 343 | ((meningiti* or meningococ* or MenB* or antimeningiti* or antimeningococ* or antiMenB*) adj vaccin*).ti,kf. |
| 344 | Poliovirus Vaccines/ or Poliovirus Vaccine, Inactivated/ or Poliovirus Vaccine, Oral/ |
| 345 | (((polio* or antipolio*) adj vaccin*) or ((IPV or OPV) and polio* and vaccin*)).ti,kf. |
| 346 | Tuberculosis Vaccines/ or BCG Vaccine/ |
| 347 | (TB vaccin* or BCG vaccin* or calmette* vaccin* or (tubercul* adj4 vaccin*)).ti,kf. |
| 348 | *Rotavirus Vaccines/ or (Rotavirus Vaccines/ and ec.fs.) |
| 349 | ((rotavir* or rota-vir* or antirotavir* or antirota-vir*) adj4 vaccin*).ti,kf. |
| 350 | *Papillomavirus Vaccines/ or *Human Papillomavirus Recombinant Vaccine Quadrivalent, Types 6, 11, 16, 18/ or ((Papillomavirus Vaccines/ or Human Papillomavirus Recombinant Vaccine Quadrivalent, Types 6, 11, 16, 18/) and ec.fs.) |
| 351 | (((HPV* or human papillomavir* or antiHPV or antipapillomavir*) adj7 vaccin*) or ((cervical cancer or anticervical cancer) adj vaccin*) or (papillomavir* and cancer and vaccin*) or (Pap* and smear? and vaccin*)).ti,kf. |
| 352 | (*Herpesvirus Vaccines/ and ec.fs.) or Chickenpox Vaccine/ or Herpes Zoster Vaccine/ |
| 353 | (((chickenpox or chicken-pox or varicella or zoster or herpeszoster or shingles) adj4 vaccin*) or ((antichickenpox or antichicken-pox or antivaricella or antizoster or antiherpeszoster or antiherpes-zoster or antishingles) adj4 vaccin*) or ZVL vaccin* or RZV vaccin*).ti,kf. |
| 354 | Smallpox Vaccine/ |
| 355 | ((smallpox or small pox or variola or antismallpox or antismall-pox or antivariola) adj4 vaccin*).ti,kf. |
| 356 | *Covid-19 Vaccines/ or chadox1 ncov-19/ or ad26covs1/ or 2019-ncov vaccine mrna-1273/ or bnt162 vaccine/ or (Covid-19 Vaccines/ and ec.fs.) |
| 357 | (((coronavir* or COVID or COVID19 or COVID2019 or 2019-nCoV or 2019nCoV or nCoV-2019 or nCoV2019 or HCoV-19 or HCoV19 or SARS-CoV-2 or SARSCo-V2 or SARS-CoV2 or SARSCoV2 or SARSCoV-2 or SARS2 or severe acute respiratory syndrome) adj4 vaccin*) or mRNA vaccines).ti,kf. |
| 358 | (Monkeypox/ or Monkeypox virus/) and Vaccines/ |
| 359 | ((monkey pox or monkeypox or antimonkey pox or antimonkeypox) adj4 vaccin*).ti,kf. |
| 360 | or/325-359 |
| 361 | 324 and 360 |

| Alternative combination of search terms - reminder interventions | |
| --- | --- |
| 362 | or/159-170,180,186-187 |
| 363 | (vaccination* or immuni#ation*).mp. |
| 364 | 362 and 363 |
| 365 | 64 and 364 |
| 366 | Reminder Systems/ec and (vaccin* or immuni#ation*).mp. |
| 367 | 361 or 365 or 366 |

| Remove low-income countries | |
| --- | --- |
| 368 | (low income countr* or Afghanistan or Burundi or Burkina Faso or Central African Republic or Eritrea or Ethiopia or Guinea or Gambia or Guinea-Bissau or Liberia or Madagascar or Mali or Mozambique or Malawi or Niger or North Korea or Korean Democratic Republic or Rwanda or Sudan or Sierra Leone or Somalia or South Sudan or Syria or Syrian Arab Republic or Chad or Togo or Uganda or Yemen or Zambia or Algeria or Angola or Bangladesh or Benin or Bhutan or Bolivia or Cote d?Ivoire or Ivory Coast or Cambodia or Cameroon or Congo or Comoros or Cabo Verde or Djibouti or Egypt or Eswatini or El Salvador or Ghana or Haiti or Honduras or India or Indonesia or Iran or Kenya or Kyrgyz Republic or Kiribati or Lao or Lebanon or Lesotho or Mauritania or Micronesia or Mongolia or Morocco or Myanmar or Nepal or Nicaragua or Nigeria or Pakistan or Papua New Guinea or Philippines or Samoa or (Sao Tome and Principe) or Senegal or Solomon Islands or Sri Lanka or Tajikistan or Tanzania or Timor-Leste or Tunisia or Ukraine or Uzbekistan or Vanuatu or Vietnam or (West Bank and Gaza) or Zimbabwe or (Africa and sub-sahara*)).ti. |
| 369 | 367 not 368 |
| 370 | limit 369 to yr="2000 -Current" |
